# Plastid Engineering for Photosynthesis‐Driven Synthesis of Hyaluronic Acid in Tobacco

**DOI:** 10.1111/pbi.70504

**Published:** 2025-12-19

**Authors:** Amanda Lopes, Omar Sandoval‐Ibáñez, Stéphanie Arrivault, David Rolo, F. Vanessa Loiacono, Alexander Erban, Daniel Karcher, Stephan Obst, Stephanie Ruf, Joachim Kopka, Ralph Bock

**Affiliations:** ^1^ Max‐Planck‐Institut für Molekulare Pflanzenphysiologie Potsdam‐Golm Germany; ^2^ Johannes Gutenberg‐Universität Mainz Institut für Organismische und Molekulare Evolutionsbiologie Mainz Germany; ^3^ Ruhr‐Universität Bochum Faculty of Biology and Biotechnology Bochum Germany; ^4^ Martin‐Luther‐University, Institute of Pharmacy Halle Germany; ^5^ Department of Experimental Plant Biology Charles University Prague Czech Republic

**Keywords:** chloroplast, hyaluronic acid, metabolic engineering, *Nicotiana tabacum*, plastid, plastid transformation, synthetic operon

## Abstract

Hyaluronic acid (HA) is a glycosaminoglycan composed of alternating units of N‐acetylglucosamine and glucuronic acid. High moisture retention, viscoelasticity and biocompatibility are unique features that make HA polymers attractive compounds for medical applications and aesthetic purposes. Current synthesis of HA polymers relies on microorganisms and requires supply of glucose in bioreactors to produce glucose‐6‐phosphate and fructose‐6‐phosphate as precursors for HA biosynthesis. By contrast, photosynthetic organisms generate glucose‐6‐phosphate and fructose‐6‐phosphate as autotrophic products of CO_2_ fixation via the Calvin‐Benson‐Bassham (CBB) cycle. Here we explored the possibility to harness chloroplast metabolism for the light‐driven production of HA in the model organism tobacco ( *Nicotiana tabacum* ). An operon of five streptococcal genes were introduced into the plastid genome of tobacco to drive HA‐synthesis by expression elements that confer low, medium or high expression levels. Photoautotrophic growth over the entire life cycle was only achieved in transplastomic lines with low transgene expression levels. Surprisingly, accumulation of HA polymers was observed only under heterotrophic growth conditions. Proteomic analysis revealed low accumulation levels of the first pathway enzyme in the transplastomic lines, and low contents of the final pathway enzyme (HA synthase) upon autotrophic growth. Altered abundances of proteins involved in photosynthesis and central metabolism were observed under autotrophic growth conditions, and metabolite profiling confirmed that photoautotrophic HA biosynthesis depleted CBB cycle derivatives and triggered plastid‐associated stress responses. Our work demonstrated the feasibility of tapping the CBB cycle for HA synthesis and identified bottlenecks for plant‐based production of carbohydrate polymers.

## 1 Introduction

Hyaluronic acid (also known as hyaluronan or hyaluronate; HA) is a glycosaminoglycan polymer that represents an essential component of the extracellular matrix of vertebrates. It provides structural support to shape tissue architecture, provide elasticity and maintain moisture levels (Abatangelo et al. 2020). In addition, HA plays a significant role as a signalling molecule in embryonic development, tissue expansion and architecture, as well as cell proliferation and motility (Abatangelo et al. 2020; Marinho et al. 2021). HA is a linear polysaccharide consisting of alternating units of N‐acetylglucosamine (GlcNAc) and glucuronic acid (GlcA) connected by β‐1,3‐ and β‐1,4‐glycosidic bonds, thus forming [−3‐GlcNAc‐1‐β‐4‐GlcA1‐β‐]_n_ disaccharide repeats (Meyer and Palmer 1934; Valachová et al. 2024). HA polymerisation is catalysed by the enzyme HA synthase (HAS) that uses UDP‐GlcNAc and UDP‐GlcA as substrates. In addition to vertebrates, HAS‐encoding genes are found in some pathogenic bacteria such as *Pasteurella multocida* , group A and group C *Streptococci*, and Chlorella virus PBCV‐1 (De Angelis et al. 1993, 1997, 1998).

In addition to its remarkably high moisture retention potential and viscoelasticity properties, HA has substantial pharmaceutical and biotechnological value due to its biocompatibility, biodegradability and lack of immunogenicity (Iaconisi et al. 2023). These attractive properties have stirred a significant interest in the development of production platforms for HA. As extraction from animal tissue waste is costly, involves harsh processes and has the risk of potential contaminations with animal proteins and viruses (Boeriu et al. 2013; Shiedlin et al. 2004), microbial fermentation has been explored as an alternative for industrial synthesis of HA. Initially, pathogenic bacteria that naturally produce HA were used for this purpose, especially group A and group C *Streptococci* that synthesise HA as part of their extracellular capsule that helps them to evade the immune system of their host. Although high yields could be achieved, these pathogenic bacteria produce harmful endotoxins that can contaminate HA preparations and represent serious concerns (Serra et al. 2023; Wei et al. 2024). To overcome these issues, microorganisms with a GRAS (generally regarded as safe) status have been genetically engineered for the heterologous production of HA and serve as the current source for industrial production (Wei et al. 2024). Another, more expensive, approach is the in vitro chemoenzymatic synthesis of HA (De Angelis et al. 2013; Li et al. 2020).

The development of a plant‐based platform for HA production is a promising approach worth exploring. Compared to other systems, production of HA in plants has significant attractions, including (i) the absence of replicating human pathogens, (ii) the synthesis at very low cost and (iii) the unlimited scale‐up possibilities (Buyel 2018). The potential of plants for heterologous production of HA was first explored in cultured tobacco cells expressing the Chlorella virus *HAS* gene with or without a vacuolar targeting signal (Rakkhumkaew et al. 2013). The transgenic cell lines synthesised HA and deposited it on the cell surface, beneath the cell wall and in subcellular fractions. Moreover, when HAS proteins were targeted to the vacuole, the cells accumulated higher amounts of HA in their vacuoles at levels reported to be > 1 mg per 10^5^ cells (Rakkhumkaew et al. 2013). Another study expressed the human *HAS2* gene in tobacco hairy roots (Nazeri et al. 2021), and achieved HA concentrations of up to 560 mg per kg fresh weight at a molecular weight > 0.8 MDa. However, expression of bacterial transgenes from the nuclear genome of eukaryotes can have certain limitations, including epigenetic transgene silencing and unwanted post‐translational protein modifications that may induce rapid protein degradation (Bolognesi and Lehner 2018; Depicker and Montagu 1997).

**FIGURE 1:**
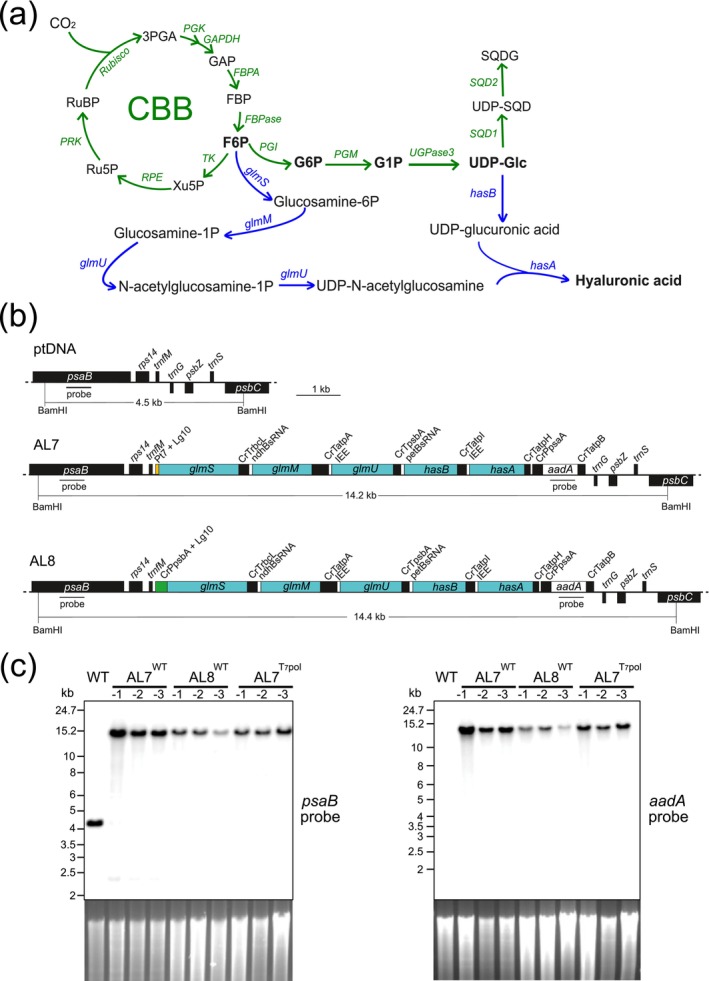
Generation of AL7^WT^, AL8^WT^ and AL7^T7pol^ transplastomic lines expressing the HA biosynthetic pathway. (a) Design of a plastid pathway for the photosynthesis‐driven production of HA using carbohydrate metabolites derived from the CBB cycle and the sulfoquinovosyl diacylglycerol biosynthesis pathway. Enzymes and the enzymatic steps derived from the CBB cycle and sulfoquinovosyl diacylglycerol biosynthesis are indicated in green. The five genes for HA biosynthesis and the enzymatic reactions catalysed by the encoded enzymes are highlighted in blue. The pathway chart indicates the two branches that produce the precursor nucleotide‐sugars UDP‐GlcNAc and UDP‐GlcA. One branch utilises F6P to synthesise UDP‐GlcNAc by three consecutive reactions mediated by glutamine‐F6P transaminase (GlmS), phosphoglucosamine mutase (GlmM), and the bifunctional UDP‐N‐acetylglucosamine diphosphorylase/glucosamine‐1‐phosphate N‐acetyltransferase (GlmU). The other branch utilises chloroplast UDP‐Glc as substrate for UDP‐glucose 6‐dehydrogenase (HasB) to synthesise UDP‐GlcA. Finally, the products from each branch are polymerised into HA by hyaluronic acid synthase (HasA). 3PGA, 3‐phosphoglycerate; CCB cycle, Calvin‐Benson‐Bassham cycle; F6P, fructose 6‐phosphate; FBP, fructose 1,6‐bisphosphate; FBPA, fructose‐1,6‐bisphosphate aldolase; FBPase, fructose‐1,6‐bisphosphatase; G1P, glucose 1‐phosphate; G6P, glucose 6‐phosphate; GAP, glyceraldehyde 3‐phosphate; GAPDH, glyceraldehyde‐3‐phosphate dehydrogenase; PGI, phosphoglucose isomerase; PGK, 3‐phosphoglycerae kinase; PGM, phosphoglucomutase; PRK, phosphoribulokinase; RPE, ribulose‐5‐phosphate 3‐epimerase; Ru5P, ribulose 5‐phosphate; RUBISCO, ribulose‐1,5‐bisphosphate carboxylase/oxygenase; RuBP, ribulose 1,5‐bisphosphate; SQD1, UDP‐sulfoquinovose synthase; SQD2, sulfoquinovosyl transferase; TK, transketolase; UDP‐GlcNAc, UPD‐N‐acetylglucosamine; UDP‐Glc, UDP‐glucose; UGPase3, UDP‐glucose pyrophosphorylase 3; Xu5P, xylulose 5‐phosphate. (b) Maps of the targeting region in the tobacco plastid genome (ptDNA; upper panel) and the modified regions in the transplastomic lines generated by stable transformation of the chloroplast genome (middle and bottom panels). Shown are the operons for HA biosynthesis driven by the T7 RNA polymerase promoter from phage T7 (Pt7) fused to the 5′ UTR of *gene10* from phage T7 (Lg10) (AL7; middle panel), or driven by the *psbA* promoter from *Chlamydomonas reinhardtii* (CrPpsbA) fused to Lg10 (AL8; bottom panel). The selectable marker gene *aadA* is driven by the *psaA* promoter from *C. reinhardtii* (CrPpsaA) and is fused to the atpB 3′ UTR from *C. reinhardtii* (CrTatpB). The 3′ UTRs from the *C. reinhardtii* plastid genes *rbcL* (CrTrbcL), *atpA* (CrTatpA), *psbA* (CrTpsbA), *atpI* (CrTatpI) and *atpH* (CrTatpH) were introduced after the individual genes of the operon. The intercistronic expression element (IEE) and analogous sequences derived from the 5′ region of *ndhB* (ndhBsRNA) and *petB* (petBsRNA) were introduced for stability and translation of the processed monocistronic transcripts. The recognition sites for digestion with the restriction enzyme BamHI used for the RFLP analysis and the expected fragment size are indicated. The binding sites of hybridisation probes for RFLP analysis are indicated as black horizontal bars below the *psaB* and *aadA* genes. (c) RFLP analysis of transplastomic tobacco lines by Southern blotting. Samples of total DNA extracted from AL7^WT^, AL8^WT^ and AL7^T7pol^ plants were digested with BamHI, and the fragments were detected by hybridisation with radiolabeled *psaB‐* or *aadA*‐specific probes (cf. panel b). Fragment sizes of the molecular weight marker are indicated for each hybridised blot. The ethidium bromide‐stained gel prior to hybridisation is shown as loading control below each blot.

The plastid (chloroplast) genome has been an attractive target for synthetic biology applications (Scharff and Bock 2014). Advantages of using plastid genome engineering technology include the precise integration of foreign DNA via homologous recombination, the absence of epigenetic transgene silencing, and the possibility of stacking multiple transgenes in synthetic operons and their expression as polycistronic transcripts (Bock 2015). The latter feature is particularly appealing for metabolic engineering approaches, which often require the expression of multiple enzymes to build a heterologous metabolic pathway in the host plant (Bock 2022). Moreover, many biosynthetic pathways take place in plastids, thus offering a plethora of precursors that can be used as building blocks and channelled into new metabolic pathways (Fuentes et al. 2018). The successful use of plastids as production factories for novel metabolites by expressing synthetic pathways from the chloroplast genome has been demonstrated, for example, with the synthesis of the precursor of the antimalarial drug artemisinin in tobacco (Fuentes et al. 2016), the synthesis of the cyanogenic glucoside dhurrin (Gnanasekaran et al. 2016) and the synthesis of the ketocarotenoid astaxanthin (Lu et al. 2017). We, therefore, hypothesised that it may be possible to also reconstitute the HA pathway in tobacco chloroplasts through plastid genome engineering. Importantly, the two precursors for HA synthesis are synthesised in chloroplasts: Fructose 6‐phosphate (F6P) is produced by the Calvin‐Benson‐Bassham (CBB) cycle, and UDP‐glucose (UDP‐Glc) is synthesised as an intermediate in the sulfoquinovosyl diacylglycerol (SQDG) biosynthetic pathway (Aarabi et al. 2023; Okazaki et al. 2009; Wenqi 2024; Figure 1a).

In this work, we selected five genes from *Streptococcus* (*glmS*, *glmM*, *glmU*, *hasB* and *hasA*) to build the biochemical pathway for synthesis of HA within plastids, relying on F6P and UDP‐Glc as precursors (Figure 1a). Testing different expression strategies, we show that HA can be synthesised in transplastomic tobacco plants. We provide a comprehensive characterisation of the transplastomic lines, including determination of their HA contents, and their changes in protein accumulation and in central metabolism under different growth conditions. Finally, our work identifies bottlenecks that limit the biosynthetic capacity for heterologous carbohydrate polymers in chloroplasts.

## 2 Results

### 2.1 Generation of Transplastomic Tobacco Plants That Express the Biosynthesis Pathway for HA From the Plastid Genome

To introduce the HA biosynthetic pathway into the plastid genome, five genes from *Streptococcus equi* subs. *zooepidemicus* ATCC 35246 (*glmS*, *glmM*, *glmU*, *hasB* and *hasA*) were selected to link the heterologous HA biosynthesis pathway via F6P and UDP‐Glc to the CBB cycle and chloroplast sulfolipid synthesis (Figure 1a). Since homologous recombination between duplicated expression elements from tobacco can lead to rearrangements and genome instability (Min et al. 2015; Rogalski et al. 2006), we used expression elements from *Chlamydomonas reinhardtii* to drive transgene expression. The five transgenes were designed to be expressed from a synthetic operon under the control of the inducible T7 RNA polymerase promoter (Pt7; construct AL7) or the constitutive *psbA* promoter from the green alga *Chlamydomonas reinhardtii* (CrPpsbA; construct AL8; Figure 1b). Processing elements were introduced between the coding sequences to generate stable monocistronic mRNAs by post‐transcriptional cleavage. The intercistronic expression element (IEE) derived from the *psbT*‐*pbsH* intergenic region (Zhou et al. 2007), which has been successfully utilised in a variety of artificial operons for metabolic engineering in plastids (Agrawal et al. 2022; Fuentes et al. 2016; Gnanasekaran et al. 2016; Lu et al. 2013, 2017), was introduced in front of the *glmU* and *hasA* coding sequences (Figure 1b). To avoid reduced accumulation of endogenous transcripts by depletion of the IEE‐binding *trans*‐factor HCF107 (Legen et al. 2018), two additional processing elements corresponding to PPR protein‐binding sites at the 5′ ends of the *ndhB* and *petB* mRNAs were introduced in front of the coding sequence of *glmM* and *hasB*, respectively (Legen et al. 2018; Figure 1b). Finally, 3′ UTR sequences derived from *Chlamydomonas reinhardtii* plastid genes were introduced after each coding sequence to ensure stable accumulation of monocistronic mRNAs (Figure 1b). The operons in constructs AL7 and AL8 were generated *in planta* by particle gun‐mediated (biolistic) co‐transformation. Recombination between the co‐transformed plasmids pAL4 and pAL5 produced the AL7 locus in the plastid genome, whereas recombination between pAL4 and pAL6 generated the AL8 locus (Figures S1 and S2).

Constructs AL7 and AL8 were introduced into wild‐type (WT) tobacco plants to produce the transplastomic lines AL7^WT^ and AL8^WT^. In addition, the AL7 construct was introduced into the Nt‐DK320 recipient line, which harbours a plastid‐encoded T7 RNA polymerase under the control of the synthetic theophylline‐responsive riboswitch (Hoelscher et al. 2022; Figure S3), to produce inducible AL7^T7pol^ lines based on the RAmpER (RNA amplification‐enhanced riboswitch) system (Emadpour et al. 2015). With this experimental setup, a range of expression strengths of the HA biosynthetic pathway was intended to be covered, with high constitutive expression in AL8^WT^ lines, medium to high expression upon theophylline induction in AL7^T7pol^ and low‐level (leaky) expression in AL7^WT^ lines (Hoelscher et al. 2022).

With each construct, several independent transplastomic lines were obtained by selection for spectinomycin resistance conferred by the *aadA* marker gene. Three lines per construct were purified to homoplasmy by conducting additional rounds of selection and regeneration under spectinomycin selection (to eliminate residual wild‐type copies of the highly polyploid plastid genome; Greiner et al. 2020), and subsequently characterised in detail. Transgene integration into the plastid genome was assessed by restriction fragment length polymorphism (RFLP) analyses via Southern blotting. To this end, DNA samples from the wild type and three independent AL7^WT^, AL8^WT^ and AL7^T7pol^ lines each were digested with the restriction enzyme BamHI, and relevant plastid DNA fragment sizes were analysed by hybridisation with probes targeting the *psaB* gene and the resistance gene *aadA* (Figure 1c). RFLP analysis employing the *psaB* probe revealed a shift in the size of the hybridising restriction fragment from 4.5 kb in the wild type to 14.4 kb in all transplastomic lines, consistent with correct integration of the AL7 and AL8 constructs by homologous recombination into the plastid genome (Figure 1b,c). Absence of the hybridisation signal for the wild‐type genome suggested a homoplasmic status of all AL7^WT^, AL8^WT^ and AL7^T7pol^ transplastomic lines. Hybridisation with the *aadA* probe detected the same fragment in the transplastomic lines and gave no signal in the wild type, as expected (Figure 1c).

### 2.2 Growth and Development of Transplastomic Plants Expressing the HA Biosynthetic Pathway

**FIGURE 2:**
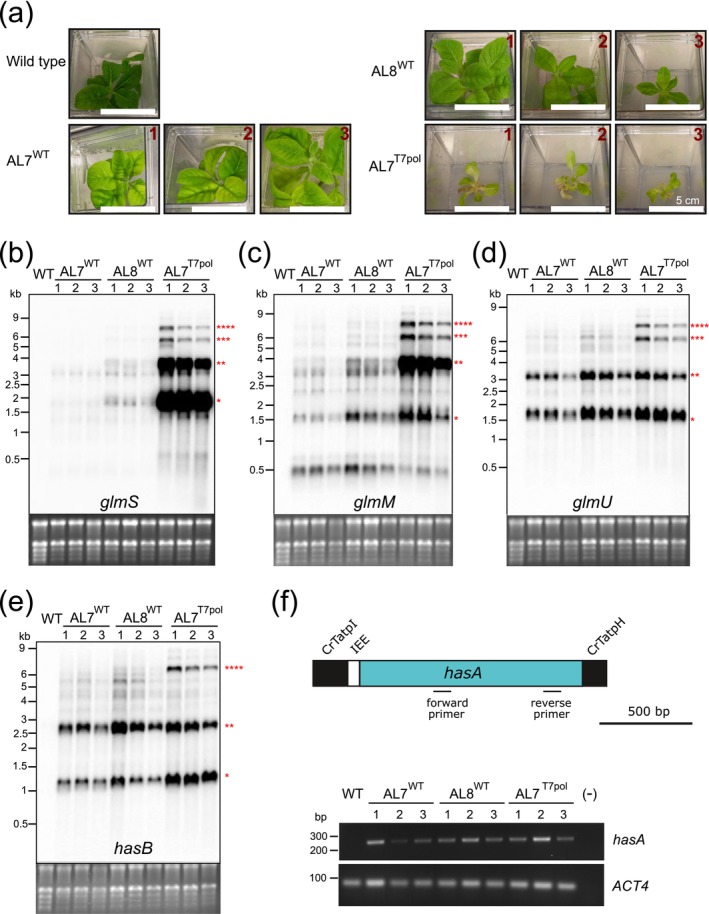
Phenotypes of AL7^WT^, AL8^WT^ and AL7^T7pol^ transplastomic lines, and accumulation of transcripts encoding the enzymes of the HA biosynthetic pathway. (a) Phenotypes of homoplasmic AL7^WT^, AL8^WT^ and AL7^T7pol^ transplastomic plants. The wild type and three independently obtained transplastomic lines were cultivated on MS medium supplemented with 3% (w/v) sucrose. For the AL7^WT^, AL8^WT^ and AL7^T7pol^ plants, the medium was additionally supplemented with spectinomycin. Scale bar: 5 cm. (b–e) Northern blot analyses to assess the accumulation for transcripts encoding the enzymes of the HA biosynthetic pathway in the AL7^WT^, AL8^WT^ and AL7^T7pol^ lines. Samples of 2 μg total RNA were resolved under denaturing conditions by gel electrophoresis. After blotting, the membranes were hybridised to radiolabeled probes specific for (b) glmS, (c) glmM, (d) *glmU* and (e) *hasB* (see Figure S6). The ethidium bromide‐stained gels prior to blotting are shown as control for equal loading below each blot. Red asterisks indicate the expected sizes for monocistronic (*), dicistronic (**), tricistronic (***) and tetracistronic (****) transcripts (cf. Figure S6). (f) Detection of the *hasA* transcript by semi‐quantitative RT‐PCR in samples from AL7^WT^, AL8^WT^ and AL7^T7pol^ plants and the wild type (WT). The upper panel shows the binding sites of the forward and reverse primers used to detect the accumulation of *hasA* by RT‐PCR. The bottom panel shows the result of the RT‐PCR assay to detect the *hasA* mRNA. RT‐PCR amplification was conducted for 28 cycles, and *ACTIN4* (*ACT4*) was employed as reference transcript to control for loading and cDNA amounts.

**FIGURE 3:**
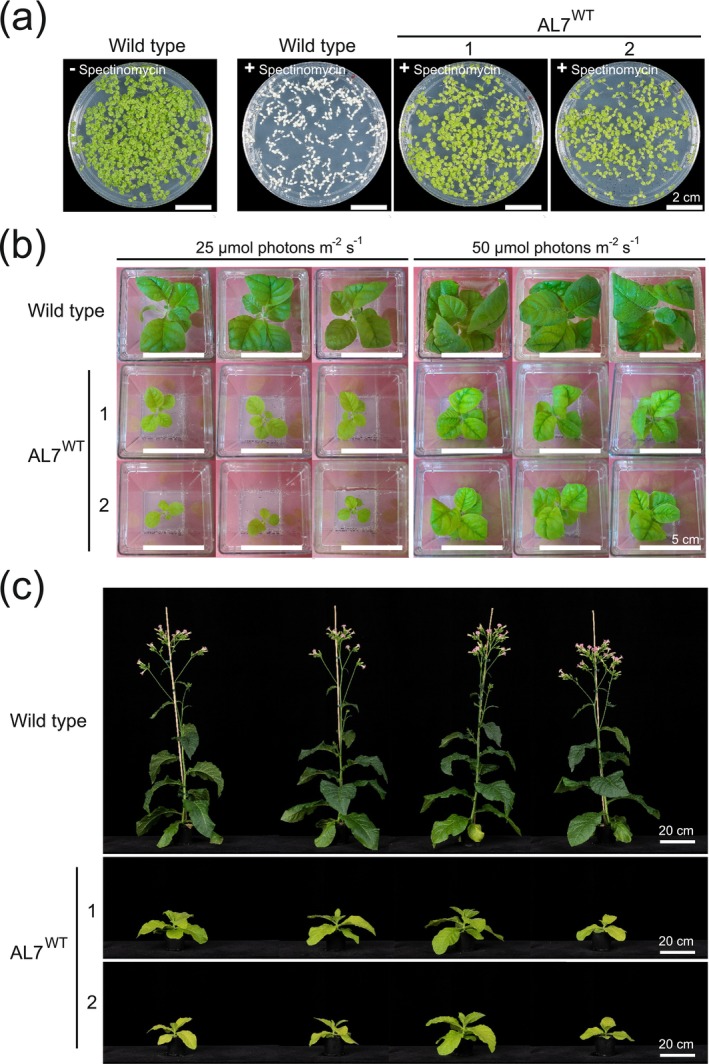
Seed tests and phenotypes of the AL7^WT^ lines cultured under heterotrophic or photoautotrophic conditions. (a) Seed test to confirm homoplasmy of AL7^WT^ lines and uniparentally maternal inheritance of the spectinomycin resistance trait. Wild type seeds and T1 seeds from two independent AL7^WT^ lines were germinated on synthetic medium with 3% (w/v) sucrose. Absence (−) or presence (+) of spectinomycin (500 mg L^−1^) is indicated. Scale bars: 2 cm. (b) Phenotype of AL7^WT^ plants in comparison to wild‐type plants upon in vitro growth on MS medium with 3% (w/v) sucrose at light intensities of 25 or 50 μmol photons m^−2^ s^−1^. The photos were taken 9 weeks after sowing. Scale bars: 5 cm. (c) Phenotype of AL^WT^ plants upon growth in soil under standard greenhouse conditions. Plants were photographed 8 weeks after sowing.

When compared to wild‐type plants, homoplasmic transplastomic plants regenerated in vitro showed pronounced phenotypes under heterotrophic cultivation conditions (Figure 2a). AL7^WT^ and AL8^WT^ plants were pale green, suggesting that expression of the HA operon affects pigment accumulation and photosynthesis (Figure 2a). When transferred to soil and grown under photoautotrophic conditions, the transplastomic plants showed strong growth retardation (Figures 3 and S4). While AL7^WT^ lines fully developed to maturity and produced viable seeds, the AL8^WT^ lines, although reaching the flowering stage, did not produce viable seeds. The AL7^T7pol^ lines (produced in the background of a plastid‐encoded T7 RNA polymerase) developed roots but showed strong growth defects already upon heterotrophic cultivation, including severe pigment deficiency, and early tissue necrosis (Figures 2a and S5a), precluding their growth under photoautotrophic conditions. Of the AL7^T7pol^ plants grown under antibiotic selection in heterotrophic conditions, two regenerated plants exhibited variegated phenotypes (Figure S5b) and one of them (AL7^T7pol^ line 1a) maintained the variegated phenotypes upon photoautotrophic cultivation (Figure S5b). However, none of the two lines produced seeds resistant to spectinomycin (Figure S5c), indicating rapid loss of the plastid transgenes when the antibiotic selection pressure was removed.

### 2.3 Transcript Accumulation and mRNA Processing of the Synthetic HA Operon in Transplastomic Tobacco Plants

Transcript accumulation from the HA operon and post‐transcriptional RNA processing were analysed by a series of northern blot experiments (Figure 2b–e). To this end, RNA samples extracted from the wild type and the AL7^WT^, AL8^WT^ and AL7^T7pol^ lines cultured under heterotrophic conditions were separated by denaturing gel electrophoresis and hybridised to probes targeting all cistrons of the operon: *glmS*, *glmM*, *glmU, hasB* and *hasA* (Figure S6). The AL7^T7pol^ lines accumulated large amounts of polycistronic and monocistronic transcripts coding for the first gene in the HA operon *glmS*. By contrast, the AL8^WT^ and AL7^WT^ lines showed much lower accumulation levels of *glmS* transcripts (Figure 2b). The accumulation of polycistronic and monocistronic mRNAs for *glmM* was similar, in that the AL7^T7pol^ transplastomic lines accumulated much higher transcript levels than the AL7^WT^ and AL8^WT^ lines (Figure 2c). The accumulation levels of the monocistronic *glmU* mRNA were slightly higher in AL8^WT^ and AL7^T7pol^ plants than in AL7^WT^ plants, but the polycistronic transcripts were much more abundant in the AL7^T7pol^ lines (Figure 2d). Hybridisation with probes targeting *hasB* revealed comparable accumulation of di‐ and monocistronic transcripts in AL7^WT^ and AL8^WT^ lines, and higher accumulation of both monocistronic and tetracistronic transcripts in the AL7^T7pol^ lines (Figure 2e). Multiple probes were designed to also analyse RNA accumulation and transcript processing patterns of the last gene in the HA operon, *hasA*. However, all of them lacked specificity for the *hasA* coding region and produced unsatisfactory results. Therefore, we assessed the abundance of the *hasA* transcript by RT‐PCR analyses. These assays readily detected *hasA* transcripts in all the transplastomic AL7^WT^, AL8^WT^ and AL7^T7pol^ lines (Figure 2f).

Since only the AL7^WT^ lines produced viable seeds, we focused our further analyses on the comparative characterisation of AL7^WT^ and wild type plants. The homoplasmic state of the AL7^WT^ lines was confirmed by seed tests, which revealed uniform resistance of the T1 progeny to spectinomycin, in line with the uniparentally maternal inheritance of the chloroplast genome (Figure 3a).

AL7^WT^ plants and wild‐type plants raised from seeds were grown in heterotrophic conditions under 25 and 50 μmol photons m^−2^ s^−1^ light intensity. Compared to the wild type, the AL7^WT^ plants exhibited delayed growth and pale green leaves under both conditions (Figure 3b), but the pigment deficiency of the AL7^WT^ plants was more severe at 25 μmol photons m^−2^ s^−1^ than at 50 μmol photons m^−2^ s^−1^ (Figure 3b). When grown under photoautotrophic conditions, AL7^WT^ plants developed more slowly and had paler leaves than wild‐type control plants (Figure 3c).

### 2.4 Transplastomic AL7^WT^ Plants Synthesise and Accumulate HA Under Heterotrophic Growth Conditions

**FIGURE 4:**
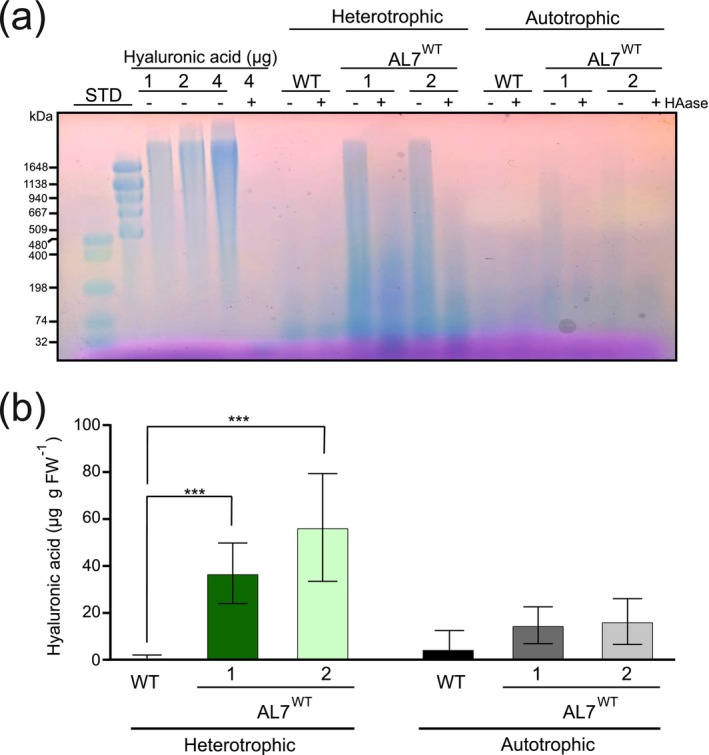
Accumulation of HA in transplastomic AL7^WT^ plants grown under heterotrophic or autotrophic conditions. (a) Qualitative assessment of the accumulation of HA in AL7^WT^ lines grown under heterotrophic or autotrophic conditions. Samples enriched in HA were treated with (+) or without (−) hyaluronidase (HAase), resolved in a 1.0% (w/v) agarose gel, and stained with the Stains‐All solution. Purified HA from *Streptococcus equi* was used as positive control. Two different standards (STD) were employed to estimate the size of the HA. WT: Wild type; *n* = 3 independent biological replicates. (b) Quantitative assessment of the accumulation of HA in AL7^WT^ lines grown under heterotrophic (green bars) or autotrophic (grey bars) conditions. HA contents were determined by the cetyltrimethylammonium bromide turbidimetric method (see Methods for details). *n* = 10 independent biological replicates.

Having isolated homoplasmic plants that harbour the HA biosynthetic operon in their plastid genomes, we next wanted to investigate whether the transplastomic plants synthesise HA. Accumulation of HA was assessed by gel electrophoresis of soluble polysaccharides extracted from leaf material of plants grown either under 50 μmol photons m^−2^ s^−1^ in heterotrophic conditions or under autotrophic conditions in the greenhouse (Figure 4a). To clearly distinguish between HA and endogenous (e.g., plant cell wall‐related) polysaccharides, all samples were analysed with and without treatment with the HA‐degrading enzyme hyaluronidase (HAase) prior to electrophoresis. The two independent AL7^WT^ lines cultivated under heterotrophic conditions exhibited high contents of HAase‐sensitive polymers, indicating synthesis and accumulation of HA (Figure 4a). Surprisingly, the accumulation of HA polymers was greatly decreased in the AL7^WT^ plants cultivated under autotrophic conditions (Figure 4a). The molecular weight (MW) of HA produced by the AL7^WT^ lines under heterotrophic conditions covered the entire range of the molecular weight marker (from 74 to 1648 kDa; Figure 4a), suggesting large polymer polydispersity. Small traces of HAase‐insensitive polysaccharides were observed in wild‐type samples under both growth conditions (Figure 4a), underscoring the importance of the HAase treatment to distinguish between HA and endogenous polysaccharides that are stained with the dye used in these assays. The HA contents in the AL7^WT^ lines were higher than those observed in AL8^WT^ and AL7^T7pol^ plants grown under heterotrophic conditions (Figure S7a).

Next, we quantitatively analysed the HA contents of plants grown under heterotrophic and autotrophic conditions by the cetyltrimethylammonium bromide turbidimetric method (Oueslati et al. 2014). The two AL7^WT^ lines grown under heterotrophic conditions exhibited HA contents of 39.9 and 61.0 μg HA per gram fresh leaf material, respectively (Figure 4b). Consistent with the gel electrophoretic analysis of HA accumulation, the amounts of HA polymers in AL7^WT^ plants grown under autotrophic growth conditions were much lower (15 and 16 μg HA g FW^−1^ in line 1 and line 2, respectively; Figure 4b). Similarly, the HA contents in AL8^WT^ and AL7^T7pol^ were much lower (9.2 and 1.3 μg HA g FW^−1^, respectively) than those observed in AL7^WT^ plants grown under heterotrophic conditions (Figure S7b).

Taken together, the results from the two different methods of HA detection and quantification (Figure 4a,b) suggest substantial HA accumulation in AL7^WT^ plants upon heterotrophic growth, but low accumulation of the polymers under autotrophic growth conditions. In addition, our data indicate impaired HA synthesis in the AL8^WT^ and AL7^T7pol^ plants as compared to AL7^WT^ plants under heterotrophic growth conditions.

### 2.5 Accumulation of HA Biosynthetic Enzymes in AL7^WT^ Plants

**FIGURE 5:**
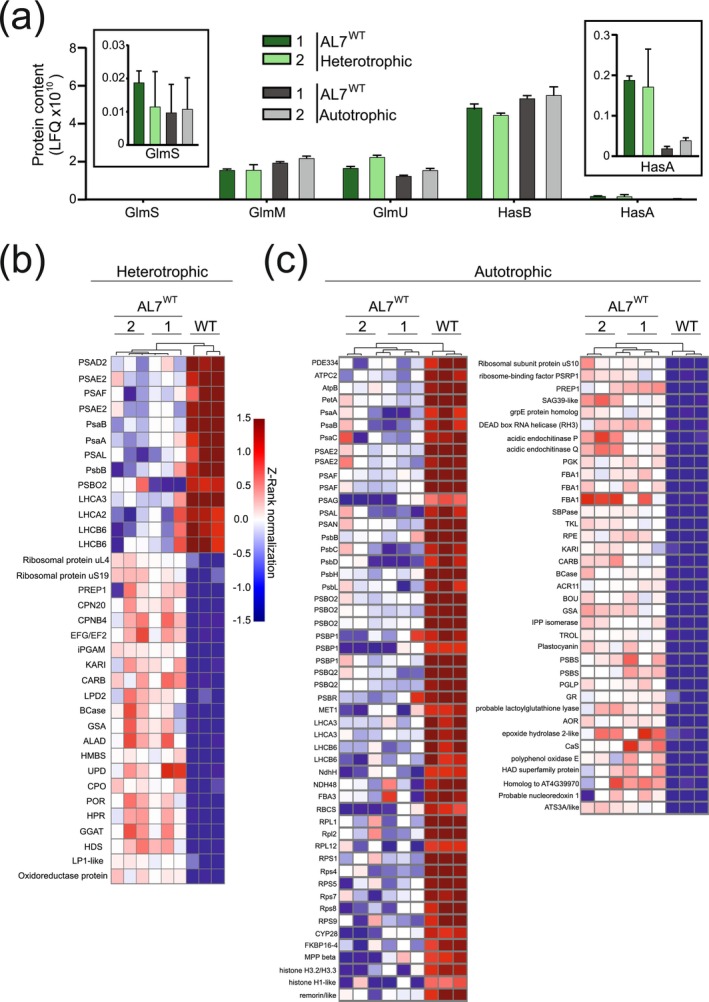
Protein profiles of AL7^WT^ lines grown under heterotrophic versus autotrophic conditions. (a) Protein accumulation of the HA biosynthetic enzymes in AL7^WT^ lines grown under autotrophic (grey bars) or heterotrophic (green bars) conditions. The y‐axis shows the log_10_ of the label‐free quantification (LFQ) values. The LFQ values for GlmS and HasA are shown as insets in the left and right corners of the panel. *n* = 3 independent biological replicates. (b, c) Z‐Rank normalisation for the protein profiles of the wild type (WT), and two independent AL7^WT^ lines grown under heterotrophic (b) or autotrophic (c) conditions. The heatmaps display only those proteins that show significant differences (Student's *t*‐test; *p* < 0.05; > 2‐fold change) in both AL7^WT^ lines compared to the wild type. The colour code indicates overaccumulation (red), no change (white) and low accumulation (blue) with respect to the median for each row. *n* = 3 independent biological replicates.

The striking difference in HA accumulation between plants grown under heterotrophic versus autotrophic conditions prompted us to analyse the accumulation of the expressed enzymes of the HA biosynthetic pathway. We performed proteomic analyses by liquid chromatography associated to tandem mass spectrometry (LC–MS/MS; Figure 5a). As expected, all AL7^WT^ lines expressed the five enzymes of the HA biosynthetic pathway. No differences were observed in the relative contents of GlmS, GlmM, GlmU and HasB between AL7^WT^ plants grown under heterotrophic and those grown under autotrophic conditions (Figure 5a; Data S1). Of these four enzymes, GlmS appears to be present at the lowest amounts (Figure 5a; Data S1). The key enzyme of the pathway, the HA synthase (HasA), was present at higher amounts in AL7^WT^ lines grown heterotrophically than in those grown autotrophically (Figure 5a; Data S1), in agreement with the higher HA contents measured in plants grown under heterotrophic conditions.

To better understand the negative effects of the plastid expression of the HA pathway on plant physiology, we also compared the accumulation profiles of endogenous proteins in the wild type and the AL7^WT^ lines under both heterotrophic and autotrophic growth conditions (Figure 5b,c; Data S2 and S3). In agreement with their pigment‐deficient leaf phenotypes, the AL7^WT^ lines showed reduced accumulation of proteins related to photosystem I (PSI), photosystem II (PSII), light‐harvesting complex I (LHCI), light‐harvesting complex II (LHCII) and also lower amounts of the presequence proteases 1 and 2 (PREP1/2) involved in transit peptide processing in chloroplasts and mitochondria (Moberg et al. 2003) under both growth conditions (Figure 5b,c). The lower accumulation of photosynthesis‐related proteins was more pronounced in the AL7^WT^ lines grown autotrophically, which additionally exhibited a decrease in protein subunits of the cytochrome *b*_6_ *f* complex (Cytb_6_f), the chloroplast ATP synthase (ATPase), the plastid‐localised NADH‐dehydrogenase‐like (NDH) complex, the PSII assembly factors CYP28 (Zhu et al. 2022) and MET1 (Bhuiyan et al. 2015), and several essential plastid‐localised ribosomal protein subunits (Tiller and Bock 2014; Figure 5c). Although the transplastomic lines exhibited pale phenotypes in both cultivation conditions, enzymes involved in the chlorophyll synthesis pathway were significantly up‐regulated exclusively in heterotrophically grown AL7^WT^ plants, and included the GLUTAMATE‐1‐SEMIALDEHYDE 2,1‐AMINOMUTASE (GSA), DELTA‐AMINOLEVULINIC ACID DEHYDRATASE (ALAD), PORPHOBILINOGEN DEAMINASE (HYDROXYMETHYLBILANE SYNTHASE, HMBS), and PROTOCHLOROPHYLLIDE OXIDOREDUCTASE (POR) (Figure 5b). The proteins upregulated in AL7^WT^ lines exclusively upon autotrophic growth corresponded to PSBS (involved in non‐photochemical quenching; Li et al. 2000), the electron carrier plastocyanin, the THYLAKOID RHODANESE‐LIKE PROTEIN (TROL) required for anchoring the ferredoxin:NADP+ oxidoreductase to the thylakoid membrane (Jurić et al. 2009), RNA helicase 3 (RH3) required for intron splicing and chloroplast ribosome assembly (Asakura et al. 2012), and the plastid‐specific ribosomal protein PSRP1 (Sharma et al. 2010; Figure 5c).

We also analysed the accumulation of proteins that may indirectly contribute to the biosynthesis of HA by providing cofactors and intermediates. Upregulation of enzymes involved in CoA biosynthesis (KETOL‐ACID REDUCTOISOMERASE, KARI), pyrimidine biosynthesis (CARBAMOYL PHOSPHATE SYNTHETASE LARGE SUBUNIT, CARB), and fatty acid synthesis (ACETYL CO‐ENZYME A CARBOXYLASE BIOTIN CARBOXYLASE SUBUNIT, BCase) were present in the AL7^WT^ lines under both cultivation conditions (Figure 5b,c). While AL7^WT^ lines accumulated large amounts of the glycolysis‐related cytoplasmic enzyme 2,3‐ BIPHOSPHOGLYCERATE‐INDEPENDENT PHOSPHOGLYCERATE MUTASE (iPGAM) upon heterotrophic cultivation (Figure 5b), autotrophic growth induced overaccumulation of CBB cycle‐related proteins, including FRUCTOSE‐BISPHOSPHATE ALDOLASE 1 (FBA1) and SEDUHEPTULOSE 1,7‐BIPHOSPHATE PHOSPHATASE (SBPase) (Figure 5c). However, low contents of the small subunit of RIBULOSE‐1,5‐BISPHOSPHATE CARBOXYLASE (RBCS) and overaccumulation of photorespiration‐related enzymes, including PHOSPHOGLYCOLATE PHOSPHATASE (PGLP; Schwarte and Bauwe 2007) and the chloroplast URIDYLYL TRANSFERASE‐LIKE protein (ACR11; Takabayashi et al. 2016), were also observed under this growth condition (Figure 5c). Finally, stress‐related proteins were upregulated in AL7^WT^ lines upon autotrophic growth, and included, for example, LACTOYLGLUTATHIONE LYASE, ALKENAL/ONE OXIDOREDUCTASE (AOR), EPOXIDE HYDROLASE 2‐LIKE, GLUTATHIONE REDUCTASE (GR) and NUCLEOREDOXIN 1 (Figure 5c). Overall, these results suggest that (i) the accumulation of the key pathway enzyme HA synthase (HasA) is strongly influenced by the growth conditions and (ii) the HA biosynthetic pathway in AL7^WT^ transplastomic lines negatively influences processes associated to photosynthesis and primary metabolism, particularly in plants that are grown autotrophically.

### 2.6 Potential Metabolic Limitations of HA Synthesis in Transplastomic AL7^WT^ Plants

**FIGURE 6:**
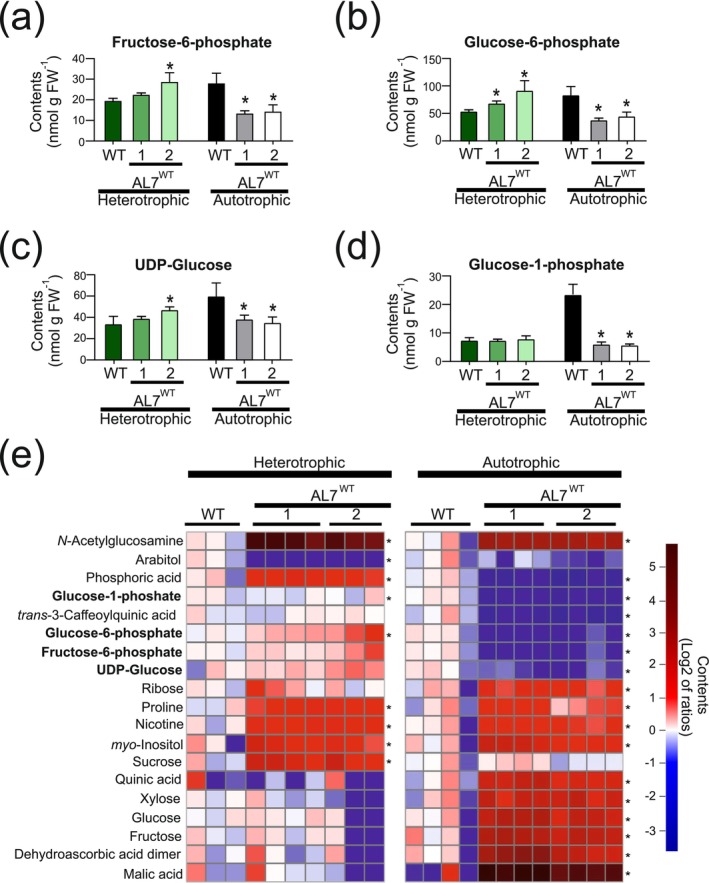
Metabolite profiles of AL7^WT^ lines grown under heterotrophic or autotrophic conditions. (a–d) Quantification of endogenous precursor metabolites of the engineered UDP‐glucuronate biosynthesis in samples from wild‐type plants (WT) and AL7^WT^ plants grown under heterotrophic (green bars) or autotrophic (grey bars) conditions. The quantification of (a) fructose‐6‐phosphate, (b), glucose‐6‐phosphate, (c) UDP‐Glucose and (d) glucose‐1‐phosphate was conducted by LC–MS/MS. The asterisks indicate significant differences between AL7^WT^ and the wild type grown under the same conditions. Statistical testing was done by the Student's *t*‐test; *p < 0.05, *n* = 3 independent biological replicates. (e) GC‐EI/TOF‐MS‐based metabolite profiling of the same paired samples from wild‐type and AL7^WT^ plants grown under heterotrophic or autotrophic conditions. The heatmaps show log_2_‐transformed mean centered ratios of overaccumulation (red), no change (white) and reduced abundances (blue). Relative changes of fructose‐6‐phosphate, glucose‐6‐phosphate, glucose‐1‐phosphate and UDP‐glucose (bold font) were co‐analysed with metabolites detected by GC‐EI/TOF‐MS. Metabolites that changed significantly and consistently in both AL7^WT^ lines cultivated either heterotrophically or autotrophically were selected. Asterisks (*) indicate fold changes > 2 or < 0.5 in both AL7^WT^ lines relative to the wild type at *p* < 0.05 (heteroscedastic Student's *t*‐test; *n* = 3–4 independent biological replicates).

The differences in protein abundances and the strong difference in HA accumulation observed in AL7^WT^ plants grown under heterotrophic versus autotrophic conditions, suggested that the biosynthesis of HA in chloroplasts suffers from metabolic limitations. To test this hypothesis and identify potential bottlenecks in the pathway, we sought to examine the accumulation of precursors and intermediates of HA biosynthesis by metabolite‐targeted LC–MS/MS analyses (Figure 6a–d). Fructose‐6‐phosphate (F6P), the common metabolic precursor of the engineered synthesis of UDP‐GlcNAc and UDP‐GlcA (Figure 1a), was found not to be depleted under heterotrophic conditions in AL7^WT^ plants compared to the wild type (Figure 6a). By contrast, F6P was significantly depleted in both transplastomic AL7^WT^ lines grown under autotrophic conditions (Figure 6a), where HA yields were substantially lower (Figure 4a,b). Both glucose‐6‐phosphate (G6P) and UDP‐Glucose (UDP‐Glc) contents correlated with F6P contents, and were significantly depleted in autotrophically cultivated AL7^WT^ lines (Figure 6b,c). By contrast, G6P and UDP‐Glc were not depleted upon heterotrophic cultivation (Figure 6b,c), and G6P accumulation was even higher in both AL7^WT^ lines than in the wild type. While glucose‐1‐phosphate (G1P) contents in both lines remained unchanged compared to the wild type under heterotrophic cultivation conditions (Figure 6d), the G1P contents in autotrophically grown AL7^WT^ lines were significantly lower than in the wild type (and similar to those observed under heterotrophic cultivation conditions; Figure 6d).

Additional classes of metabolites were analysed by GC‐EI/TOF‐MS‐based multi‐targeted metabolome profiling of primary and small specialised metabolites (Figure 6e; Data S4). An initial principal component analysis suggested substantial metabolic reprogramming in response to expression of the HA pathway and the growth conditions. The first principal component (accounting for 40.01% of the total variance) clearly distinguished autotrophically grown from heterotrophically grown AL7^WT^ lines, whereas the second principal component (accounting for 20.8% of the total variance) distinguished the autotrophically grown wild type from both cultivation conditions of the AL7^WT^ lines (Figure S8). In line with our previous analyses, the activity of the HA pathway had different effects on plant metabolism depending on the mode of cultivation. For a more comprehensive overview of the metabolic changes triggered by HA synthesis under the two growing conditions, we merged the datasets obtained from LC–MS/MS analysis and GC‐EI/TOF‐MS analysis (Figure 6e). GC‐EI/TOF‐MS did not detect glucosamine‐6‐phosphate (GlcN‐6P) or glucosamine‐1‐phosphate (GlcN‐1P), the first specific intermediates of the engineered pathway towards UDP‐GlcNAc. The method also did not identify non‐acetylated glucosamine (GlcN). Nonetheless, the activities of the GlmS, GlmM and GlmU enzymes in vivo was unambiguously demonstrated by the accumulation of substantial amounts of N‐acetylglucosamine (GlcNAc) in autotrophically grown AL7^WT^ plants and even larger amounts in heterotrophically cultivated plants (Figure 6e). In contrast to the presence of non‐polymerised GlcNAc, free glucuronic acid (GlcA) was not detected by our GC‐EI/TOF‐MS profiling of leaf material from the AL7^WT^ lines.

Along with information about the metabolite pools related to the engineered pathways (Figure 1a), the GC‐EI/TOF‐MS data provided evidence of substantial pleiotropic reprogramming of autotrophic metabolism in the AL7^WT^ lines. By contrast, the heterotrophically grown AL7^WT^ lines showed fewer changes in metabolites (*p* < 0.05 heteroscedastic Student's *t*‐test; x‐fold change > 2 or < 0.5; Figure 6e). In addition to GlcNAc, the AL7^WT^ plants overaccumulated, for example, proline, nicotine, sucrose, as well as phosphate, but were depleted for arabitol when cultivated heterotrophically (Figure 6e). Under autotrophic conditions, fructose, glucose, xylose, ribose, *myo*‐inositol, malic acid, quinic acid and nicotine were found to be increased in AL7^WT^ plants relative to the wild type, whereas chlorogenic acid (i.e., 3‐*trans*‐caffeoylquinic acid, a conjugate of quinic acid) and phosphate contents were decreased. F6P, G6P and UDP‐Glc amounts were also decreased significantly, but the change was close to the threshold of our stringency criteria (Figure 6a–d).

## 3 Discussion

In this work, we have explored the potential of chloroplasts to produce the high‐value carbohydrate polymer hyaluronic acid. Our data show that five bacterial genes expressed as an artificial plastid operon are sufficient to trigger the synthesis of HA in tobacco chloroplasts. Of the transplastomic lines generated in this study, the AL7^T7pol^ lines displayed the highest mRNA accumulation levels, even in absence of chemical induction of the RAmpER with theophylline (Figure 2b–f). This is likely attributable to the inherent leakiness of the system, in that the T7 promoter driving the HA operon is also recognised by the endogenous bacteriophage‐type RNA polymerase of the chloroplast (nucleus‐encoded plastid polymerase, NEP; Agrawal et al. 2022; Emadpour et al. 2015; Hoelscher et al. 2022). This explanation gains further support from the observed accumulation of transcripts in the AL7^WT^ lines, which occurs in the absence of the T7 RNA polymerase (Figure 2b–f).

The homoplasmic AL7^T7pol^ and AL8^WT^ lines exhibited deleterious phenotypes. Both sets of lines did not produce viable seeds, and the AL7^T7pol^ lines were not even capable of sustained photoautotrophic growth after transfer to soil. Although variegated AL7^T7pol^ lines survived under photoautotrophic growth conditions, all seeds obtained from these plants were sensitive to spectinomycin, indicating that release of the selection pressure upon growth in soil favoured the propagation of cells with wild‐type chloroplasts (Figure S5). In general, the severity of the growth phenotypes in homoplasmic lines was correlated to the expression strength of the HA operon (Figure 2). The AL7^WT^ lines, which have the lowest expression level of the operon, grew photoautotrophically and produced seeds. However, they were delayed in growth and displayed a pale‐green phenotype, suggesting that their expression strength of the HA operon is close to the maximum that can be tolerated.

The AL7^WT^ lines produced substantial amounts of HA under heterotrophic conditions, with a wide range of polymer sizes and maximum contents of 60 μg HA per gram of fresh leaf biomass. This accumulation level is lower than the highest value reported for expression in *Agrobacterium rhizogenes* ‐transformed hairy roots (of approximately 400 μg per g of wet tissue; Nazeri et al. 2021). However, the water content in cultured wet hairy root tissue tends to be higher than in greenhouse‐grown leaves, suggesting that the expression value published for hairy roots would be significantly lower, if expressed on a per unit fresh weight basis. Be that as it may, our stably transformed transplastomic tobacco plants offer an attractive production platform for HA, in that the polymer can be extracted from leafy biomass that is free of contaminating bacteria—a feature that is particularly important for applications of HA in medicine (Iaconisi et al. 2023).

Growth phenotypes and pigment‐deficient leaves have been observed previously in transplastomic studies that attempted the expression of metabolic enzymes (Bohmert‐Tatarev et al. 2011; Lössl et al. 2003; Lu et al. 2017). Taken together with these published data, our results obtained from the characterisation of primary metabolism in the transplastomic HA‐synthesising plants generated in this study suggest that the biochemical networks operating in primary metabolism are rather tightly regulated and, when tapped by a heterologously introduced pathway, respond sensitively by showing metabolic disturbances and phenotypic alterations.

A somewhat surprising finding made in the course of this work was the striking difference in HA yield between autotrophically and heterotrophically grown plants. A reasonable explanation for the substantially higher HA levels obtained under heterotrophic (or, more precisely, mixotrophic) conditions could be that the feeding of additional sugar alleviates metabolic limitations from the excessive use of F6P (produced by the CBB cycle; Figure 1) for HA synthesis. However, our proteomic analyses showed that the accumulation of the key pathway enzyme, the transmembrane protein HasA (HA synthase), was strongly decreased under autotrophic growth conditions, possibly suggesting that multiple factors contribute to the observed difference in HA synthesis capacity under autotrophic versus heterotrophic conditions. Whether the lower contents of the membrane‐anchored HasA under autotrophic growth conditions are due to protein instability and/or reduced levels of protein synthesis, is currently unknown.

Consistent with the pale‐green phenotype of our transplastomic HA‐producing plants, our proteomic data revealed reduced abundance of thylakoid proteins related to photosynthetic electron transfer. This could be due to disturbed biogenesis of thylakoidal protein complexes due to membrane integration of HasA and/or depletion of the UDP‐Glc pool by HA synthesis, which would result in impaired biosynthesis of the essential thylakoid sulfolipid SQDG (Gnanasekaran et al. 2016; Okazaki et al. 2009).

Our metabolite analyses revealed depletion of F6P, G6P, UDP‐Glc and G1P in AL7^WT^ transplastomic lines upon autotrophic growth. The depletion of these intermediates of UDP‐GlcA biosynthesis is in line with the lower HA accumulation under photoautotrophic conditions. While GlcNAc was detected in both growth conditions, UDP‐GlcNAc and GlcNAc‐1‐phosphate were not detectable by GC‐EI/TOF‐MS profiling. We hypothesise that the methoxyamination of carbonyl moieties inherent to this profiling technique converts these metabolites to a chemical derivative that is indistinguishable from GlcNAc. It also can be expected that UDP‐GlcA is unstable under the selected analysis conditions and is converted to the same derivative as GlcA.

The observed differences in the metabolite profiles of autotrophically grown plants also included metabolites associated to plant stress responses such as nicotine (Mahmoudi et al. 2025; Steppuhn et al. 2004) and proline (Ghosh et al. 2022). The increase of specific primary metabolites such as *myo*‐inositol and fructose indicates altered sucrose metabolisation, which also has been associated with stress (Saddhe et al. 2021). By contrast, heterotrophically grown AL7^WT^ lines accumulate sucrose, which can be interpreted as an early indicator of metabolic limitations (Mathan et al. 2021; Sakr 2023) caused by heterologous HA biosynthesis.

Overall, our study provides a plant‐based platform to produce HA by plastid metabolic engineering. Under heterotrophic (mixotrophic) growth conditions, the presence of sucrose in the medium favours the accumulation of HA in the transplastomic lines, likely by alleviating the carbohydrate limitation that results from impaired photosynthesis. This hypothesis is supported by the increased accumulation of G6P, which may be attributable to sucrose metabolisation and anaplerosis of CBB cycle‐derived reactions that fuel a plethora of biosynthetic pathways. By contrast, the low contents of F6P, G6P, G1P and UDP‐Glc observed in autotrophically grown AL7^WT^ lines suggest depletion of these metabolites, which was found to be correlated with lower production levels of HA, and overaccumulation of both stress‐related proteins and metabolites associated with stressful conditions. HasA was the only pathway enzyme that showed a pronounced reduction in abundance under autotrophic growth conditions. Thus, reduced autotrophic HA synthesis may be caused by depletion of UDP‐GlcA precursors (and UDP‐Glc limitation), and reduced accumulation of the final pathway enzyme HA synthase. Whether or not the latter is due to reduced rates of synthesis or increased protein turnover, remains to be determined. However, it seems possible that depletion of CBB cycle–derived metabolites and/or accumulation of intermediates in the HA synthesis pathway are responsible for most of the proteomic and metabolomic changes observed in the AL7^WT^ plants.

In summary, our results reported here suggest that the main bottlenecks of HA production in plastids are (i) the expression level of the HA synthase, (ii) the provision of precursors by the CBB cycle and the competition with sulfolipid synthesis for intermediates and (iii) the stress responses resulting from the depletion of crucial compounds in primary metabolism. Future efforts will target these limitations to further enhance the production of HA and other valuable compounds derived from plastid primary metabolism, for example, by refined metabolic pathway engineering approaches and/or the use of improved inducible transgene expression systems.

## 4 Experimental Procedures

### 4.1 Plant Material and Growth Conditions

*Nicotiana tabacum* cv. Petit Havana wild‐type (WT) plants and the previously generated transplastomic line Nt‐DK320 (Hoelscher et al. 2022) were employed as recipient lines for the plastid transformation experiments. For cultivation under aseptic conditions, seeds were surface sterilised with 2% (v/v) sodium hypochlorite, rinsed five times with sterile water for 10 min each, and stratified at 4°C for 2 days. The seeds were then sown on Murashige and Skoog (MS) medium (Murashige and Skoog 1962) supplemented with 3% (w/v) sucrose and incubated for germination and seedling growth under long‐day conditions (16 h light/8 h darkness) at light intensities of 25 or 50 μmol photons m^−2^ s^−1^, a day temperature of 25°C and a night temperature of 20°C. For autotrophic growth, seeds were germinated and cultivated in the greenhouse under long‐day conditions (16 h light/8 h darkness) at an average light intensity of 300 μmol photons m^−2^ s^−1^ and average day and night temperatures of 25°C and 20°C, respectively.

### 4.2 Construction of the glmS‐glmM‐glmU‐hasB‐hasA Operon

The *glmS*, *glmM*, *glmU*, *hasB* and *hasA* coding sequences from *Streptococcus equi* subs. *zooepidemicus* ATCC 35246 (NC_017582.1) were codon‐optimised for plastid gene expression (Table S1) and acquired by chemical gene synthesis (Invitrogen GeneArt; GeneCust). The synthetic operon was assembled under the control of the T7 RNA polymerase promoter or the plastid *psbA* promoter by recombination between plasmids pAL4 and pAL5, or between plasmids pAL4 and pAL6. Each pair of plasmids harboured 950 bp of overlapping sequence for homologous recombination. The plasmid pairs were generated using the intermediate plasmids pDK349 or pDK410 harbouring the T7 RNA polymerase promoter or the plastid *psbA* promoter (Agrawal et al. 2020; Hoelscher et al. 2022; Strand et al. 2023; Figures S1 and S2) employing the primers listed in Table S2. See Methods S1 for detailed experimental procedures.

### 4.3 Plant Transformation and Isolation of Homoplasmic Transplastomic Lines

Constructs AL7 and AL8 were generated *in planta* by biolistic co‐transformation. Recombination between co‐transformed plasmids pAL4 and pAL5 produced the AL7 locus in the plastid genome, recombination between pAL4 and pAL6 generated the AL8 locus. The AL7^WT^ and AL8^WT^ lines were generated by using wild‐type *N. tabacum* cv. Petit Havana as recipient for plastid transformation. The AL7^T7pol^ transplastomic lines were generated using the *N. tabacum* Nt‐DK320 line as recipient (Hoelscher et al. 2022). For plastid transformation, young leaves from aseptically grown tobacco plants were bombarded using the biolistic protocol (Ruf and Bock 2011). Briefly, detached leaves of the recipient lines were bombarded with DNA‐coated gold particles (20 μg of vector mix per transformation) using the DuPont PDS‐1000/He biolistic gun and hepta adapter (Bio‐Rad, Munich, Germany). The bombarded leaves were cut into small pieces (~4 × 4 mm) and placed onto MS‐based shoot regeneration medium containing modified vitamins (Duchefa M0245), 3% (w/v) sucrose, 1 mg L^−1^ 6‐benzylaminopurine and 0.1 mg L^−1^ 1‐naphthaleneacetic acid. The medium was supplemented with 500 mg L^−1^ spectinomycin for selection. Primary spectinomycin‐resistant shoots were subjected to a second round of regeneration on spectinomycin‐containing medium and homoplasmy of the lines was assessed by restriction fragment length polymorphism (RFLP) analysis via Southern blotting. Homoplasmic shoots were transferred to MS‐based rooting medium containing 3% (w/v) sucrose and supplemented with 500 mg L^−1^ spectinomycin. Rooted plantlets were then transferred to soil and grown under photoautotrophic conditions to maturity. T1 seeds obtained from transplastomic lines were surface sterilised with chlorine gas, and selected in MS medium supplemented with 3% (w/v) sucrose and 500 mg L^−1^ spectinomycin to test for inheritance of the antibiotic resistance trait.

### 4.4 Nucleic Acid Extraction, Gel Electrophoresis and Nucleic Acid Detection

For total DNA extraction, leave material was snap frozen in liquid nitrogen and ground to a fine powder. Total DNA was extracted using a cetyltrimethylammonium bromide‐based extraction method (Doyle and Doyle 1990). The frozen ground material was suspended in 2 volumes of CTAB buffer supplemented with RNase A and heated at 65°C for 30 min. After extraction with chloroform/isoamyl alcohol (24:1 v/v), the DNA was precipitated from the aqueous phase and washed with 100% (v/v) isopropanol and 70% (v/v) ethanol, respectively. The nucleic acids were redissolved in water and quantified using a Thermo Scientific NanoDrop spectrophotometer.

Total RNA was extracted from frozen ground material using the TRIzol reagent (Thermo Fisher Scientific) following the manufacturer's instruction, and quantified using a Thermo Scientific NanoDrop spectrophotometer.

For RFLP analyses, samples of 3 μg total DNA were digested with the restriction enzyme BamHI and the resulting fragments were resolved by electrophoresis in 0.7% (w/v) agarose gels. For blotting, the gel was sequentially treated with (i) 0.25 M HCl for 15 min and rinsed with distilled water, (ii) 0.5 M NaOH for 30 min and rinsed with distilled water, (iii) 0.5 M NaOH and 1.5 M NaCl for 30 min and rinsed with distilled water, (iv) and incubated in 1 M Tris, 3 M NaCl (pH 6.5) for 15 min. The DNA fragments were then transferred to Hybond‐XL membranes (Cytiva Life Sciences Amersham) by capillary blotting using 10 × SSC buffer [1.5 M NaCl, 0.15 M trisodium citrate], and UV‐crosslinked at 0.12 J cm^−2^ (BLX‐254 Crosslinker, Vilber Lourmat). For hybridisation, [α‐^32^P]‐dCTP‐labelled *aadA* and *psaB* probes were produced from purified PCR products using the primer pairs oBock4‐oBock5, and oBock104‐oBock105, respectively (Table S2). After overnight hybridisation at 65°C in Church & Gilbert's hybridisation buffer [1% (w/v) bovine serum albumin (BSA), 1 mM ethylenediaminetetraacetic acid tetrasodium salt dihydrate (Na_2_EDTA), 7% (w/v) sodium dodecyl sulfate (SDS), 0.5 M Na_2_HPO_4_, pH 7.2], the membranes were washed with 2 × SSC buffer supplemented with 0.1% (w/v) SDS for 20 min at room temperature, and then washed twice in 0.5 × SSC buffer supplemented with 0.1% (w/v) SDS for 15 min at 65°C. Finally, the membranes were placed in a storage phosphor screen (GE Healthcare) for overnight exposure, and the image of the screen was obtained with a Typhoon TRIO + scanner (GE Healthcare).

For northern blot analyses, samples of 2.5 μg total RNA were denatured at 75°C for 15 min in MOPS buffer [20 mM 3‐(N‐morpholino)propanesulfonic acid, 5 mM sodium acetate, 1 mM Na_2_EDTA, pH 7.0] containing 18% (v/v) formaldehyde, 46% (v/v) formamide and 0.06 mg L^−1^ ethidium bromide, and then resolved by electrophoresis in 1% (w/v) denaturing agarose gels [MOPS buffer with 1% agarose and 16% (v/v) formaldehyde]. The resolved RNA samples were transferred to Hybond‐XL membranes (Cytiva Life Sciences Amersham) by capillary blotting using 5 × SSC buffer, and UV‐crosslinked twice at 0.12 J cm^−2^ (BLX‐254 Crosslinker, Vilber Lourmat). [α‐^32^P]‐UTP‐labelled RNA probes targeting the transcripts of *glmS*, *glmM*, *glmU* and *hasB* were generated by in vitro transcription using the MAXIscript T7 Transcription Kit (Invitrogen). After overnight hybridisation at 65°C in Church & Gilbert's hybridisation buffer, the membranes were washed sequentially in 1 × SSC with 0.2% (w/v) SDS, 0.5 × SSC with 0.2% (w/v) SDS, and 0.5 × SSC with 0.2% (w/v) SDS at 65°C. Finally, the membranes were placed in a storage phosphor screen (GE Healthcare) for overnight exposure, and images were acquired with a Typhoon TRIO + scanner (GE Healthcare).

For RT‐PCR analyses, RNA samples were treated with DNase (TURBO DNA‐free Kit, Invitrogen) and reverse transcribed to cDNA using SuperScript III Reverse Transcriptase (Invitrogen) according to the manufacturer's protocol. RT‐PCR was performed using specific primers (Table S2) and DreamTaq DNA Polymerase (Thermo Scientific) following the manufacturer's instructions.

### 4.5 Extraction of Hyaluronic Acid and Alcohol Insoluble Residues

Hyaluronic acid extraction was conducted according to Nazeri et al. (2021) with modifications. Briefly, leaf material was ground in liquid nitrogen and the powder was suspended in extraction buffer [0.1 M NaNO_3_, 5 mM NaEDTA, 5 mM cysteine, and 10 mM dithiothreitol (DTT)] to a concentration of 200 mg fresh weight (FW) per mL buffer. The samples were then centrifuged at 21 000 *g* for 20 min at room temperature. The recovered soluble fraction was mixed with 1.5 volumes of absolute ethanol, incubated on ice for 3 h, and then centrifuged at 21 000 *g* for 20 min at 4°C. The pellet was washed by centrifugation with 100% (v/v) ethanol and then with 100% (v/v) acetone, and dried overnight at room temperature. The pellet was resuspended in 0.1 M NaNO_3_, adjusting the final concentration to 5 g FW mL^−1^, and mechanically disrupted with a plastic pistil. After incubation for 3 h at room temperature, the samples were centrifuged at 21 000 *g* for 20 min at room temperature. Finally, the aqueous fraction containing alcohol insoluble residues (AIR; including soluble cell wall‐derived components and HA) was collected and employed for HA quantification and polymer characterisation.

### 4.6 Hyaluronic Acid Quantification and Detection via Gel Electrophoresis

Quantification of HA was conducted using the cetyltrimethylammonium bromide turbidimetric method (CTM) adapted from Oueslati et al. (2014). AIR samples (22.5 μL) and HA standard (22.5 μL) each of a dilution series of 4, 2, 1, 0.5, 0.25 and 0 mg mL^−1^ HA from *Streptococcus equi* (Sigma‐Aldrich) were mixed with 6 μL of phosphate buffer (80 mM sodium phosphate pH 7, 308 mM NaCl), and treated with or without hyaluronidase (0.5 μg μL^−1^; Type I‐S, Sigma‐Aldrich). The samples were then incubated at 37°C for 1 h, mixed with 1 volume of 0.1 M phosphate buffer (pH 7.0), and further incubated at 37°C for 15 min. For quantification of HA, 1 volume of 37°C pre‐warmed CTM reagent [2.5% (w/v) CTAB, 2% (w/v) NaOH] was added to the samples and the standards treated with or without hyaluronidase, and mixed at 300 rpm for 10 s. The absorbance was detected at 600 nm using a CLARIOstar microplate reader (BMG LABTECH). The standard curves and the HA contents were determined by the difference of the absorbances between samples treated and not treated with hyaluronidase.

The in‐gel detection of HA was conducted according to the Echelon Biosciences Inc. protocol for HA gel electrophoresis adapted from Cowman et al. (2011). AIR samples and HA standards treated with or without hyaluronidase were mixed with sample buffer [0.02% bromophenol blue, 2 M sucrose in 1 × TAE (Tris‐Acetate‐EDTA) buffer, pH 8.3] and resolved by electrophoresis in a 1% (w/v) agarose gel (pre‐incubated overnight in 1 × TAE buffer). The gel was incubated overnight with Stains‐All solution [0.005% (w/v) Stains‐All (Sigma‐Aldrich) in 50% (v/v) ethanol] and de‐stained with 30% (v/v) ethanol. The HA ladders Select‐HA HiLadder and LoLadder (Echelon Bioscience) were employed to estimate the polymer size of HA in the AIR samples.

### 4.7 Protein Extraction and Quantification by LC–MS/MS

Protein extraction was conducted according to Sandoval‐Ibáñez et al. (2022). Leaf samples were ground in liquid nitrogen and extracted with TKMES buffer [100 mM Tricine‐KOH pH 7.5, 10 mM KCl, 1 mM MgCl_2_, 1 mM EDTA, 10% (w/v) sucrose, 0.2% (v/v) Triton X‐100, 1 mM DTT, 2 × protease inhibitor cocktail (cOmplete Protease Inhibitor Cocktail EDTA‐free, Roche)].

Total protein extracts were quantified using the Pierce Coomassie (Bradford) Protein Assay Kit, and samples of 40 μg were resolved in the first 1.5 cm of an SDS‐polyacrylamide gel. The gel pieces were subjected to in‐gel trypsin digestion according to Shevchenko et al. (2006) with modifications.

Proteomic analyses were conducted according to Rolo et al. (2024), and protein identification and quantification were performed with MaxQuant version 1.6.0.13 (Cox and Mann 2008).

The Perseus software version 2.1.3.0 (Tyanova et al. 2016) was used for statistical analysis and processing of LFQ intensity data. Relevant changes in protein accumulation were selected by significance (*p*‐value < 0.05, two‐sample Student's *t*‐test) and a greater or equal to 2‐fold change of LFQ abundance, comparing the transplastomic lines to the WT under the same cultivation conditions. See Methods S2 for detailed experimental procedures.

### 4.8 Hexose Phosphate and UDP‐Glucose Extraction and Targeted Quantification by LC–MS/MS

Fructose‐6‐phosphate (F6P), glucose‐6‐phosphate (G6P), UDP‐glucose (UDP‐Glc) and glucose‐1‐phosphate (G1P) were quantified according to Arrivault et al. (2009, 2015).

Targeted quantification was performed with a Dionex HPLC system coupled to a Finnigan TSQ Quantum Discovery MS‐Q3 (Thermo Scientific) equipped with an electrospray (ESI) interface, operated in the negative ion mode with selected reaction monitoring (SRM), an ion spray voltage of 4000 V and a capillary temperature of 320°C. Prior to injection, a mixture of stable isotopically labelled compounds of known concentrations was added to the standards and extracts to correct for matrix effects (Arrivault et al. 2015). LC–MS/MS SRM peaks were integrated using the ThermoFinnigan processing software package LCQuan‐2.5. Metabolites were quantified by comparing the integrated signal peak area with the calibration curves obtained with authentic standards. See Methods S3 for detailed experimental procedures.

### 4.9 Metabolite Profiling Analyses by GC–MS (GC‐EI/TOF‐MS)

Equal volumes of residual samples after metabolite‐targeted quantifications by LC–MS/MS were dried and analysed by a gas chromatography–mass spectrometry (GC–MS) method designed for the multi‐targeted profiling of polar metabolite fractions that are enriched in primary and small specialised metabolites as described by Fiehn et al. (2000) with modifications reported by Erban et al. (2020). Metabolite annotation was performed manually supervised by matching mass spectra and retention indices (RIs) to the reference data of the Golm Metabolome Database (Hummel et al. 2013). Abundance data of arbitrary units were normalised by sample fresh weight (mg FW) and maximum scaled (%) per analytical feature prior to statistical procedures. Relevant changes in metabolite accumulation were selected by significance (*p*‐value < 0.05, two‐sample Student's *t*‐test) comparing the transplastomic lines to the wild type under the same cultivation conditions. See Methods S4 for detailed experimental procedures.

## Author Contributions

A.L., O.S.‐I., J.K. and R.B. designed the experiments. A.L., O.S.‐I., S.A., D.R., F.V.L., A.E., D.K. and J.K. conducted the experiments and analysed the data. R.B. secured funding. A.L., O.S.‐I., J.K. and R.B. wrote the paper with input from all co‐authors.

## Funding

This work was supported by the Alexander von Humboldt Foundation, Bonn, Germany. Coordination of Superior Level Staff Improvement (CAPES), Brazil. Max‐Planck‐Gesellschaft.

## Disclosure

*Accession Numbers:* The plasmid sequences were deposited in GenBank (https://www.ncbi.nlm.nih.gov/genbank/) under accession numbers PX367283 for pAL4, PX367284 for pAL5, and PX367285 for pAL6.

## Conflicts of Interest

The authors declare no conflicts of interest.

## Supporting information


**Figure S1:** Physical maps of plasmids pDK394 and pDK410.
**Figure S2:** Plasmid maps of the constructs generated to introduce the HA biosynthetic pathway into the chloroplast.
**Figure S3:** Physical map of the targeting region in the plastid genome of the transplastomic recipient line Nt‐DK320.
**Figure S4:** Phenotype of transplastomic AL8WT lines upon growth under photoautotrophic conditions.
**Figure S5:** Phenotype of transplastomic AL7T7pol lines upon growth under photoheterotrophic and photoautotrophic conditions.
**Figure S6:** Identification of monocistronic and polycistronic transcripts synthesised from the HA operon in the transplastomic lines.
**Figure S7:** Accumulation of HA in homoplasmic AL7WT, AL8WT and AL7T7pol lines grown under photoheterotrophic conditions.
**Figure S8:** Principal component analysis of samples analysed by GC‐EI/TOF‐MS‐based metabolite profiling.
**Table S1:** Codon‐optimised sequences of glmS, glmM, glmU, hasB and hasA for plastid expression.
**Table S2:** List of primers used for cloning, preparation of hybridisation probes and RT‐PCR analyses.
**Methods S1:** Construction of the glmS‐glmM‐glmU‐hasB‐hasA operon.
**Methods S2:** Protein extraction and quantification by LC–MS/MS.
**Methods S3:** Hexose phosphate and UDP‐glucose extraction and targeted quantification by LC–MS/MS.
**Methods S4:** Metabolite profiling analyses by GC–MS (GC‐EI/TOF‐MS).

**Data S1:** List of LFQ intensity, sequence coverage and peptide count of the 5 enzymes of the HA biosynthesis pathway.
**Data S2:** List of proteins downregulated and upregulated in AL7WT lines 1 and 2 growing under heterotrophic conditions.
**Data S3:** List of proteins downregulated and upregulated in AL7WT lines 1 and 2 growing under autotrophic conditions.
**Data S4:** List of metabolites significantly downregulated and upregulated in AL7WT lines 1 and 2 grown under autotrophic and heterotrophic conditions.

## Data Availability

The data that supports the findings of this study are available in the Supporting Information of this article.
