# Supplementary material for: Plastid Engineering for Photosynthesis‐Driven Synthesis of Hyaluronic Acid in Tobacco
Source: Plant Biotechnol J. 2025 Dec 19;24(4):2541–58. doi: 10.1111/pbi.70504 (PMC13140457; doi:10.1111/pbi.70504)
Supplement: Supplementary file 1 — Figure S1: Physical maps of plasmids pDK394 and pDK410. Figure S2: Plasmid maps of the constructs generated to introduce the HA biosynthetic pathway into the chloroplast. Figure S3: Physical map of the targeting region in the plastid genome of the transplastomic recipient line Nt‐DK320. Figure S4: Phenotype of transplastomic AL8WT lines upon growth under photoautotrophic conditions. Figure S5: Phenotype of transplastomic AL7T7pol lines upon growth under photoheterotrophic and photoautotrophic conditions. Figure S6: Identification of monocistronic and polycistronic transcripts synthesised from the HA operon in the transplastomic lines. Figure S7: Accumulation of HA in homoplasmic AL7WT, AL8WT and AL7T7pol lines grown under photoheterotrophic conditions. Figure S8: Principal component analysis of samples analysed by GC‐EI/TOF‐MS‐based metabolite profiling. Table S1: Codon‐optimised sequences of glmS, glmM, glmU, hasB and hasA for plastid expression. Table S2: List of primers used for cloning, preparation of hybridisation probes and RT‐PCR analyses. Methods S1: Construction of the glmS‐glmM‐glmU‐hasB‐hasA operon. Methods S2: Protein extraction and quantification by LC–MS/MS. Methods S3: Hexose phosphate and UDP‐glucose extraction and targeted quantification by LC–MS/MS. Methods S4: Metabolite profiling analyses by GC–MS (GC‐EI/TOF‐MS). [file PBI-24-2541-s002.pdf]

Supporting information for:

Plastid engineering for photosynthesis-driven synthesis of hyaluronic acid in tobacco

By Amanda Lopes, Omar Sandoval-Ibáñez, Stéphanie Arrivault, David Rolo, F. Vanessa Loiacono, Alexander Erban, Daniel Karcher, Stephan Obst, Stephanie Ruf, Joachim Kopka, Ralph Bock

This file contains the following items:

Supporting figures

Figure S1: Physical maps of plasmids pDK394 and pDK410.

Figure S2: Plasmid maps of the constructs generated to introduce the HA biosynthetic pathway into the chloroplast.

Figure S3: Physical map of the targeting region in the plastid genome of the transplastomic recipient line Nt-DK320.

Figure S4: Phenotype of transplastomic AL8^{WT} lines upon growth under photoautotrophic conditions.

Figure S5: Phenotype of transplastomic AL7^{T7pol} lines upon growth under photoheterotrophic and photoautotrophic conditions.

Figure S6: Identification of monocistronic and polycistronic transcripts synthesized from the HA operon in the transplastomic lines.

Figure S7: Accumulation of HA in homoplasmic AL7^{WT}, AL8^{WT} and AL7^{T7pol} lines grown under photoheterotrophic conditions.

Figure S8: Principal component analysis of samples analyzed by GC-EI/TOF-MS-based metabolite profiling.

Supporting tables

Table S1: Codon-optimized sequences of *glmS*, *glmM*, *glmU*, *hasB*, and *hasA* for plastid expression.

Table S2: List of primers used for cloning, preparation of hybridization probes, and RT-PCR analyses.

Supporting experimental procedures

Methods S1: Construction of the glmS-glmM-glmU-hasB-hasA operon.

Methods S2: Protein extraction and quantification by LC-MS/MS.

Methods S3: Hexose phosphate and UDP-glucose extraction and targeted quantification by LC-MS/MS.

Methods S4: Metabolite profiling analyses by GC-MS (GC-EI/TOF-MS).

Supporting references

Supporting datasets are provided in a separate excel file, and include:

Data S1: List of LFQ intensity, sequence coverage, and peptide count of the 5 enzymes of the HA biosynthesis pathway.

Data S2: List of proteins downregulated and upregulated in AL7WT lines 1 and 2 growing under heterotrophic conditions.

Data S3: List of proteins downregulated and upregulated in AL7WT lines 1 and 2 growing under autotrophic conditions.

Data S4: List of metabolites significantly downregulated and upregulated in AL7WT lines 1 and 2 grown under autotrophic and heterotrophic conditions.

Supporting figures

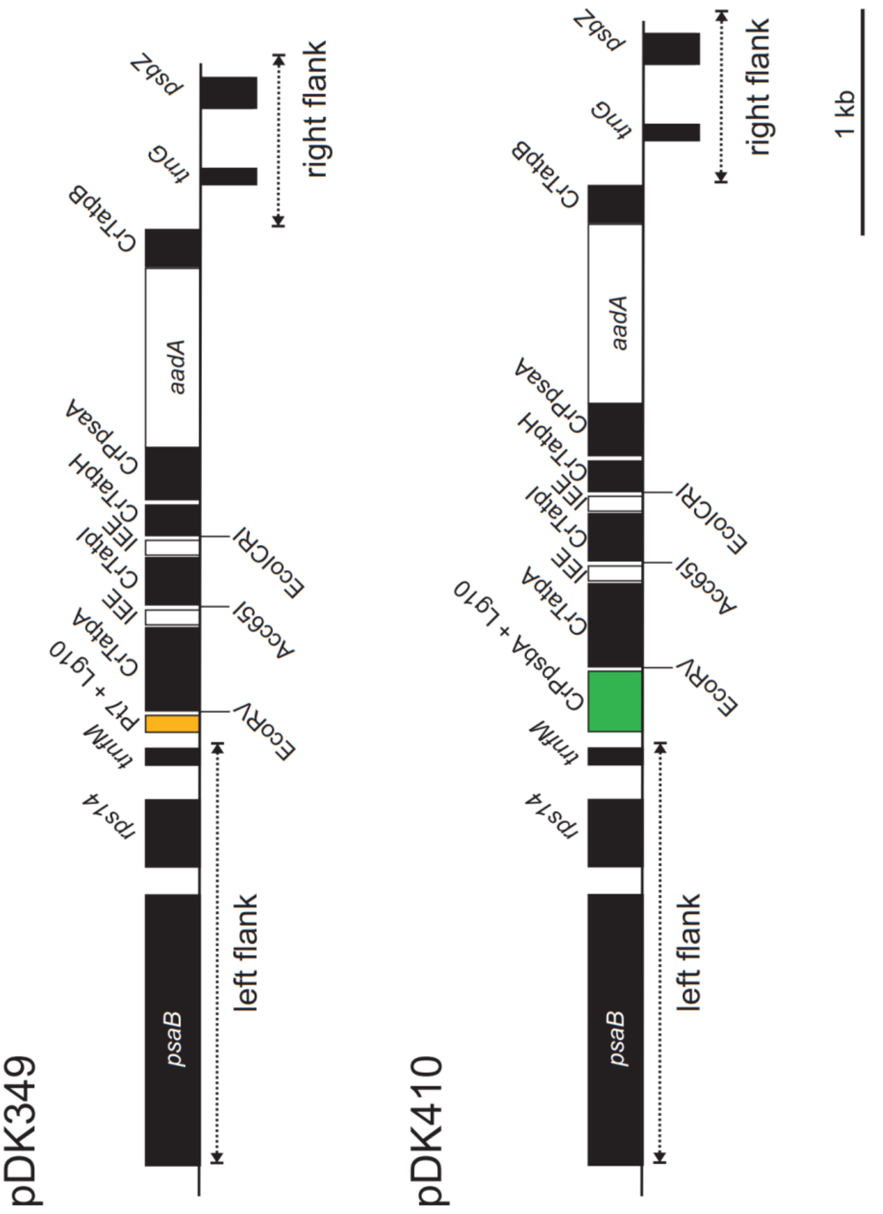

Figure S1. Physical maps of plasmids pDK349 and pDK410. Vectors pDK349 and pDK410 are derived from pRB98. The plasmids pDK349 and pDK410 contain the promoters Pt7 and CrPpsbA, respectively. Both intermediate plasmids include the terminators of *atpA*, *atpI*, and *atpH* from *Chlamydomonas reinhardtii*, intercistronic expression elements (IEE), and the *aadA* cassette under the control of the *psaA* promoter and the *atpB* terminator from *Chlamydomonas reinhardtii*.

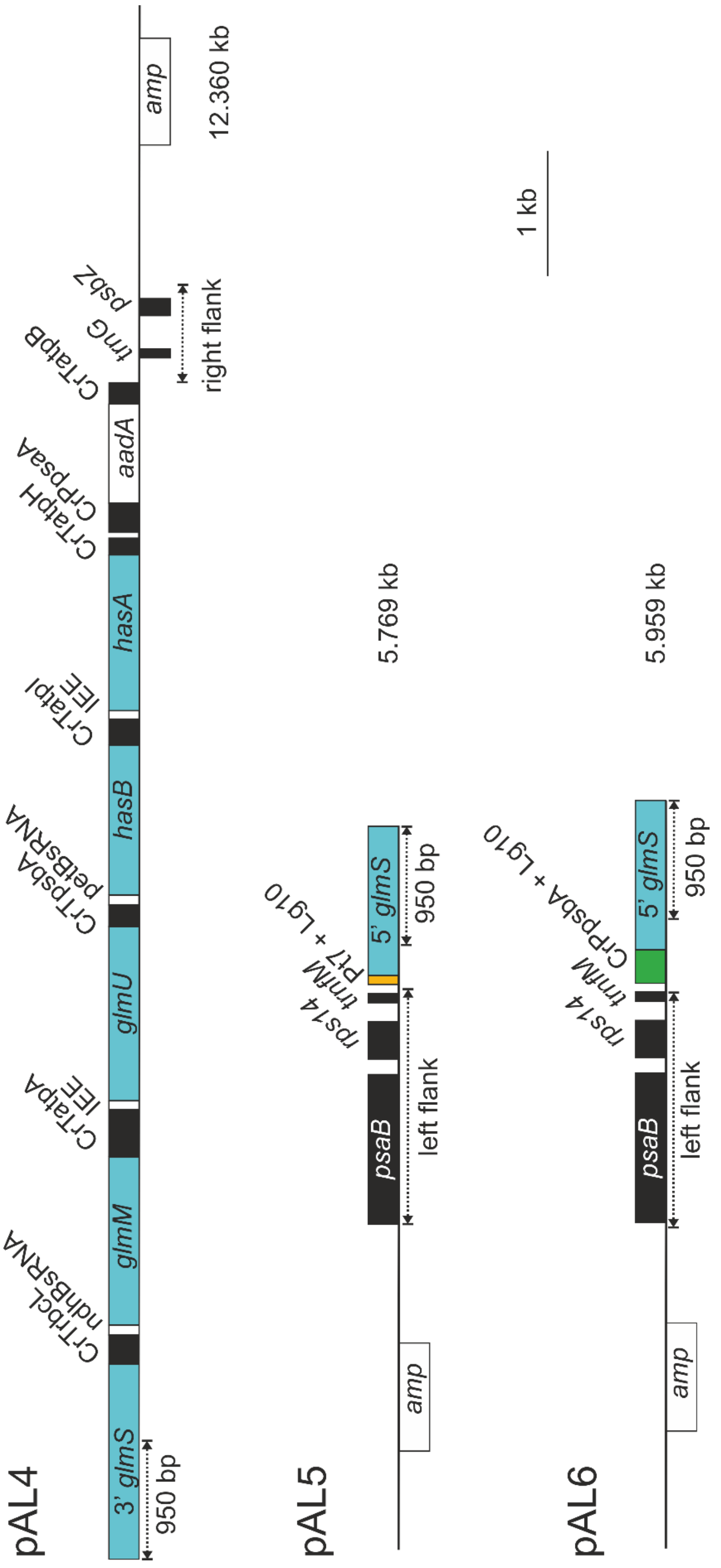
pAL4
3' glmS
950 bp
CrTrbcL
ndhBsRNA
glmM
CrTatpA
IEE
glmU
CrTpsbA
petBsRNA
hasB
CrTatpI
IEE
hasA
CrTatpH
CrPpsaA
aadA
CrTatpB
trnG
psbZ
right flank
amp
12.360 kb
pAL5
amp
psaB
left flank
rps14
trnfM
PT7 + Lg10
5' glmS
950 bp
5.769 kb
1 kb
pAL6
amp
psaB
left flank
rps14
trnfM
CrPpsbA + Lg10
5' glmS
950 bp
5.959 kb

Figure S2. Plasmid maps of the constructs generated to introduce the HA biosynthetic pathway into the chloroplast. Vector pAL4 contains the 3' part of the *glmS* gene, the other four genes involved in the HA biosynthetic pathway, the selectable marker gene *aadA*, and the right flanking region for integration into the plastid genome by homologous recombination. Plasmids pAL5 and pAL6 contain promoters driving the HA operon (Pt7 and CrPpsbA, respectively), the 5' part of *glmS* (including a stretch of 950 bp overlapping with the *glmS* sequence in pAL4 to facilitate *in vivo* reconstitution of the full operon by homologous recombination) and the left flanking region for integration into the plastid genome by homologous recombination. A mix of pAL4 and pAL5 was used to co-transform the wild type and the Nt-DK320 recipient line, generating $AL7^{WT}$ and $AL7^{T7pol}$ transplastomic plants (cf. Figure 1). $AL8^{WT}$ transplastomic lines were produced by co-transforming wild-type tobacco with a mix of vectors pAL4 and pAL6.

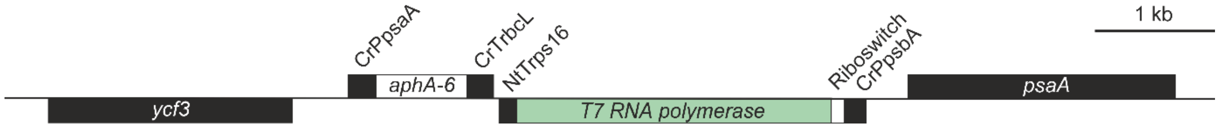

Figure S3. Physical map of the targeting region in the plastid genome of the transplastomic recipient line Nt-DK320.The Nt-DK320 line was previously obtained and described in detail (Hoelscher et al. 2022). It harbors the T7 RNA polymerase gene inserted between the *ycf3* and *psaA* genes and controlled by the theophylline-responsive riboswitch. The chimeric *aphA-6* gene serves as selectable marker conferring kanamycin resistance. CrPpsaA, *C. reinhardtii psaA* promoter; CrPpsbA, *C. reinhardtii psbA* promoter; CrTrbcL, *C. reinhardtii rbcL* 3’ UTR; NtTrps16: tobacco *rps16* 3’ UTR.

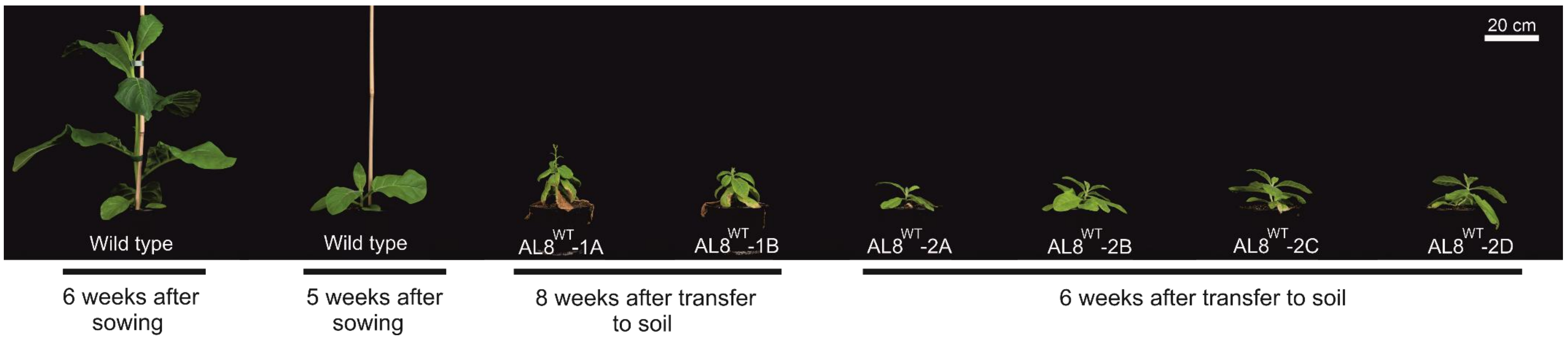

20 cm
Wild type
Wild type
AL8^WT-1A
AL8^WT-1B
AL8^WT-2A
AL8^WT-2B
AL8^WT-2C
AL8^WT-2D
6 weeks after sowing
5 weeks after sowing
8 weeks after transfer to soil
6 weeks after transfer to soil

Supplementary Figure 4. Phenotype of transplastomic AL8^{WT} lines upon growth under photoautotrophic conditions. Three independently generated AL8^{WT} lines were rooted *in vitro* prior to transfer to soil. Two plants from line 1, and four from line 2 were transferred and grown under standard greenhouse conditions (16 h light / 8 h darkness; average light intensity: 300 µmol photons m^{-2} s^{-1}; average day temperature: 25 °C; average night temperature: 20 °C).

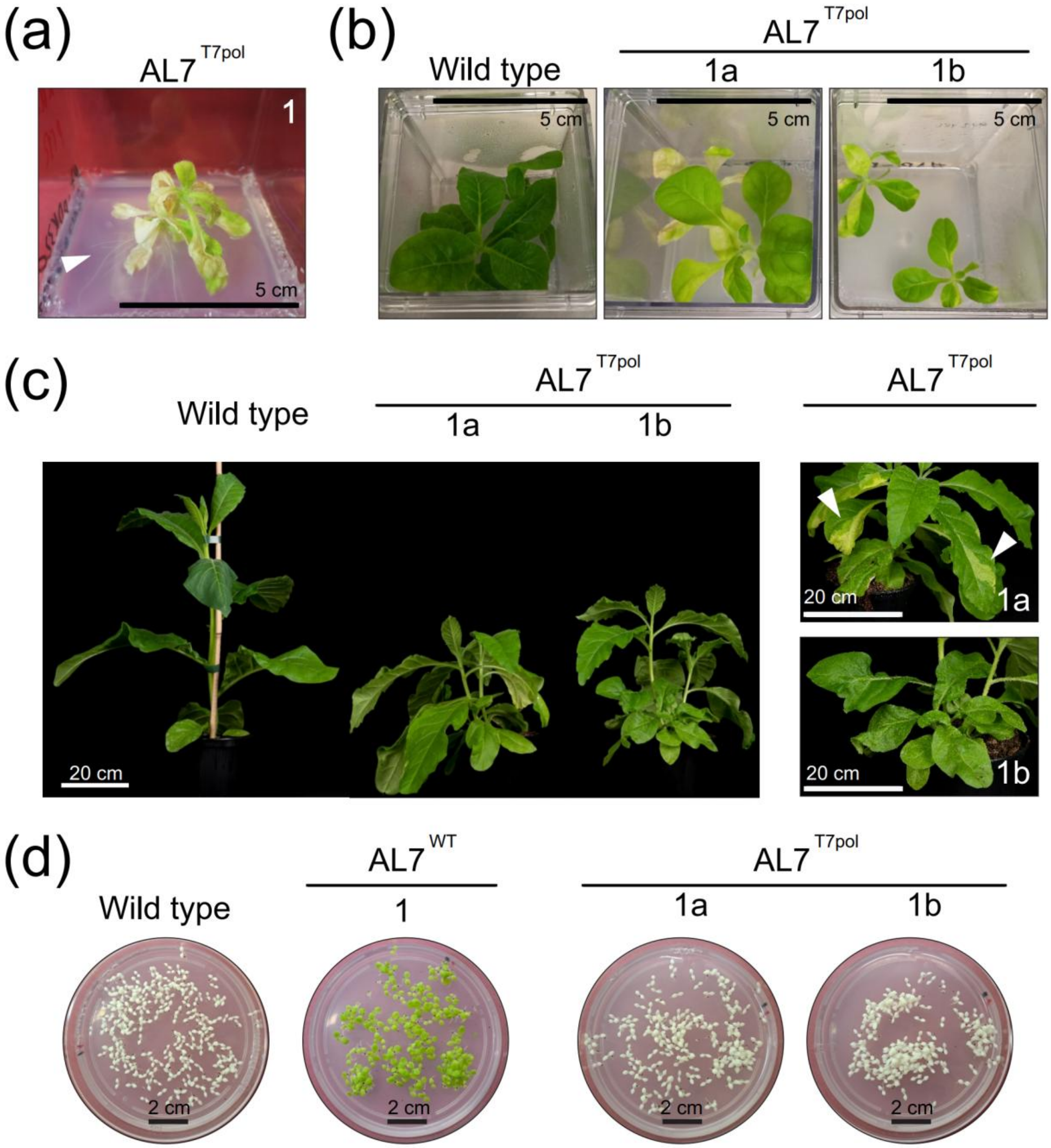

Figure S5. Phenotype of transplastomic AL7^{T7pol} lines upon growth under photoheterotrophic and photoautotrophic conditions. (a) Homoplasmic AL7^{T7pol} line grown under heterotrophic conditions. The arrowhead indicates root formation upon *in vitro* growth of regenerated shoots. (b) Images of two independently regenerated AL7^{T7pol} lines (1a and 1b) displaying variegated phenotypes. (c) Images of the two independently regenerated AL7^{T7pol} lines upon growth in soil. Plantlets rooted *in vitro* were transferred to soil and grown under standard greenhouse conditions (16 h light / 8 h darkness; average light intensity: 300

μmol photons m^{-2} s^{-1}; average day temperature: 25 °C; average night temperature: 20 °C). (d) Seeds test to analyze homoplasmy of $AL7^{T7pol}$ lines and uniparentally maternal inheritance of the spectinomycin resistance trait. Wild-type seeds and T1 seeds from the two independent $AL7^{T7pol}$ lines were germinated on synthetic medium with 3% (w/v) sucrose in the presence of spectinomycin (500 mg L^{-1}). Seeds from a homoplasmic $AL7^{WT}$ line served as positive control.

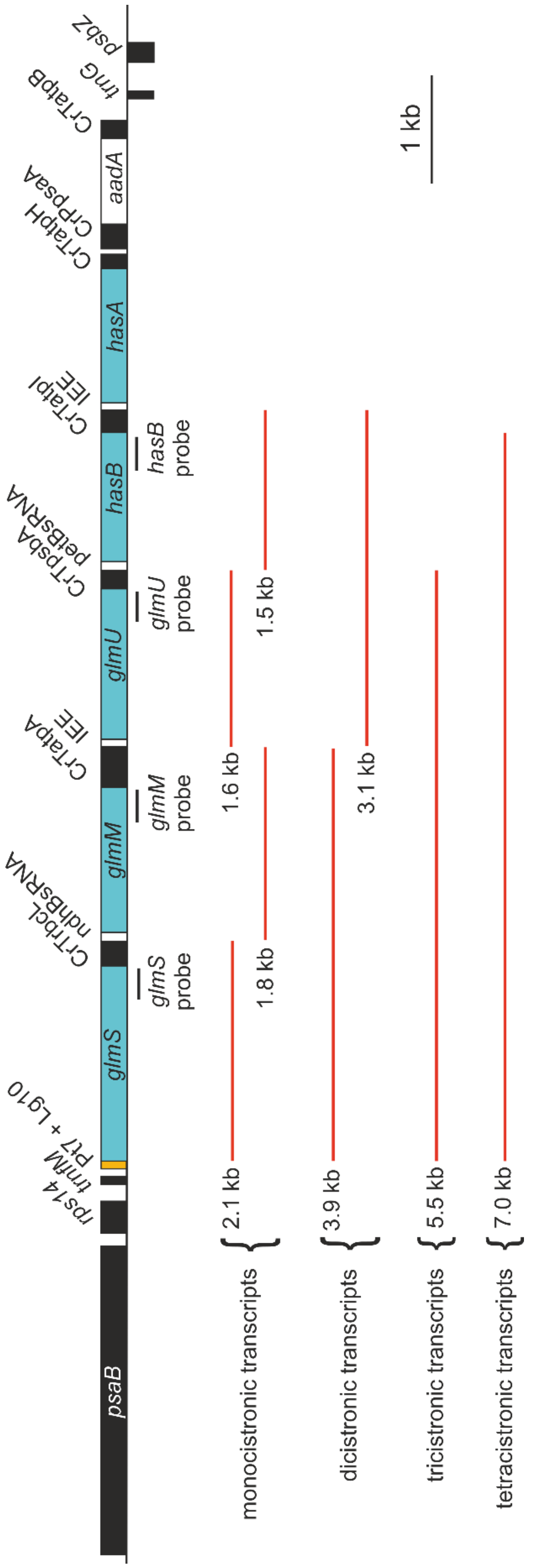

psaB
rps14
trnfM
P17 + Lg10
glmS
CrTrbcL
ndhBsRNA
glmM
CrTatpA
IEE
glmU
CrTpsbA
petBsRNA
hasB
CrTatpI
IEE
hasA
CrTatpH
CrPpsaA
aadA
CrTatpB
trnG
psbZ
glmS probe
glmM probe
glmU probe
hasB probe
monocistronic transcripts
2.1 kb
1.8 kb
1.6 kb
1.5 kb
dicistronic transcripts
3.9 kb
3.1 kb
tricistronic transcripts
5.5 kb
tetracistronic transcripts
7.0 kb
1 kb

Figure S6. Identification of monocistronic and polycistronic transcripts synthesized from the HA operon in the transplastomic lines. The positions of the hybridization probes used to detect the transcripts derived from the *glmS*, *glmM*, *glmU* and *hasB* genes are indicated as horizontal bars below the genes. Major RNA species detected by northern blot analyses are indicated as red horizontal bars and their sizes are given in kb.

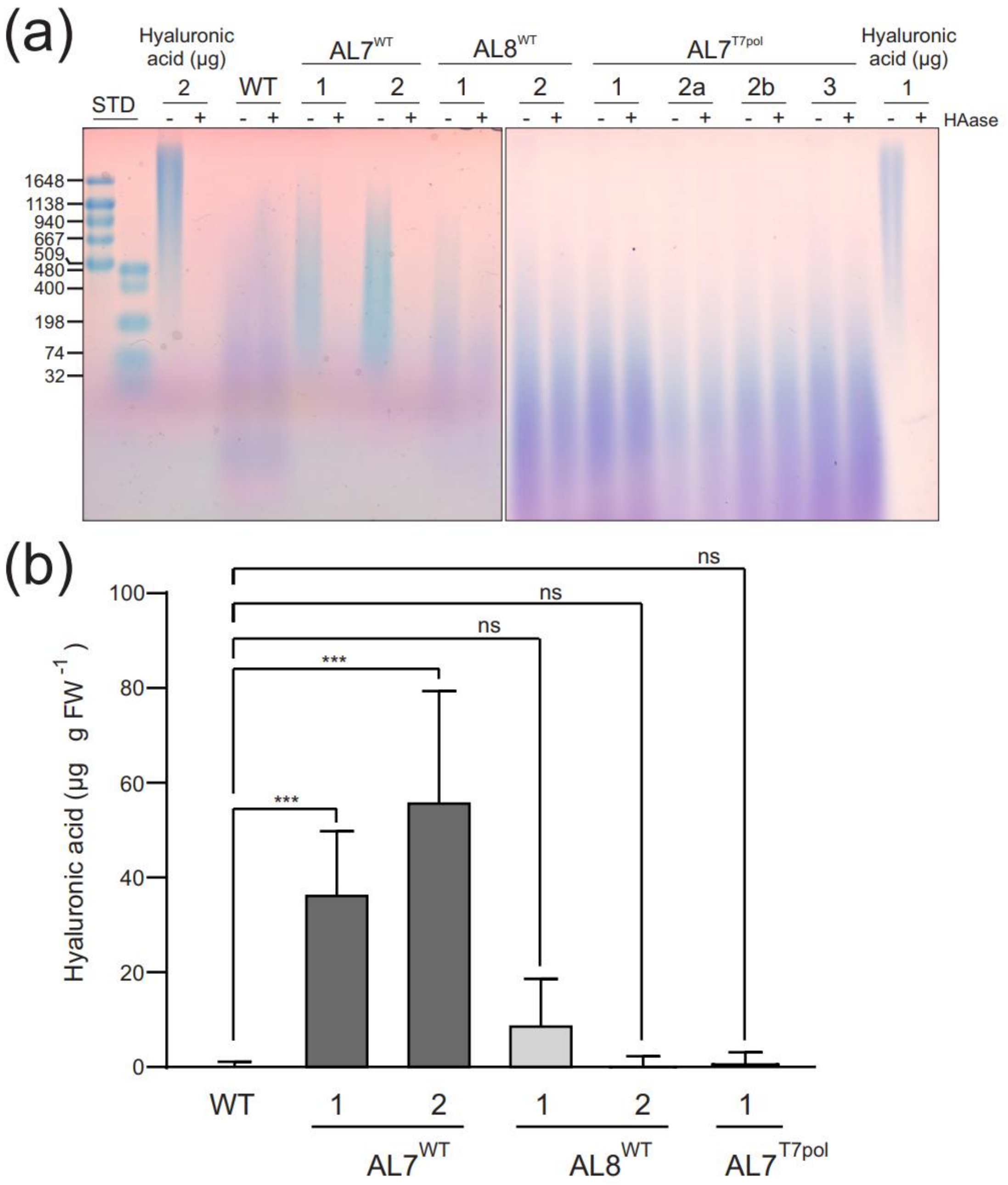

Figure S7. Accumulation of HA in homoplasmic AL7^{WT}, AL8^{WT} and AL7^{T7pol} lines grown under photoheterotrophic conditions. (a) Qualitative assessment of the accumulation of HA in AL7^{WT}, AL8^{WT} and AL7^{T7pol} lines grown under heterotrophic conditions. Samples enriched in HA were treated with (+) or without (-) hyaluronidase (HAase), resolved in a 1.0% (w/v) agarose gel, and stained with the Stains-All solution. Purified HA from *Streptococcus equi* was used as positive control. Two different standards (STD) were employed to estimate the size of the HA. WT: wild type; n=1-2 biological replicates. (b) Quantitative assessment of the accumulation of HA in AL7^{WT}, AL8^{WT} and AL7^{T7pol} lines grown under heterotrophic conditions.

HA contents were determined by the cetyltrimethylammonium bromide turbidimetric method (see Methods for details). n=2-13 independent biological replicates.

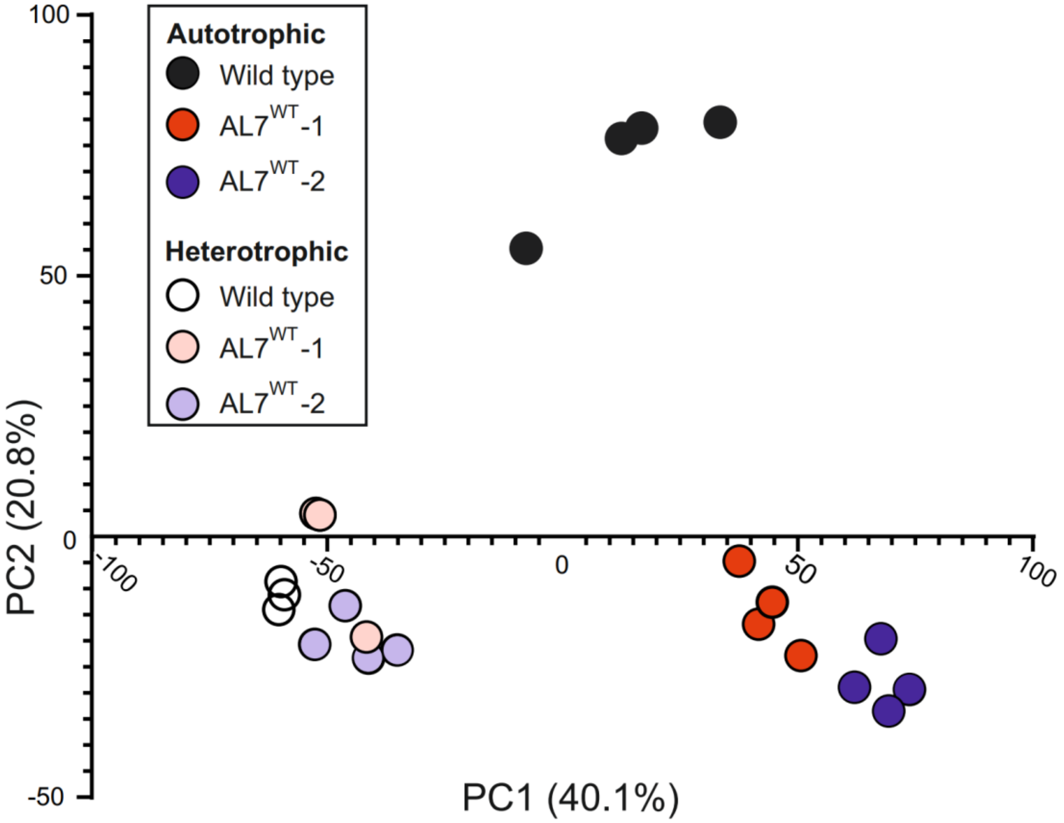

Figure S8. Principal component analysis of samples analyzed by GC-EI/TOF-MS-based metabolite profiling. The figure shows the distribution of samples characterized by non-targeted metabolite profiling by principal component 1 (PC1; x-axis) and principal component 2 (PC2; y-axis). Samples were from the wild type (WT; black and white circles), $AL7^{WT}$ line 1 (red and pink circles), and $AL7^{WT}$ line 2 (dark blue and light blue circles) grown under photoautotrophic or heterotrophic conditions.

Supporting tables

Table S1. Codon-optimized sequences of *glmS*, *glmM*, *glmU*, *hasB*, and *hasA* for plastid expression.

Gene	Codon-optimized sequence (5'-3')
glmS	tttgtttaactttaagaaggagatatagatATGTGTGGAATTGTTGGAGTTGTTGGTTCTAGAAATGCTACTGATA<br>TTTTAATGCAAGGATTAGAAAAATTAGAATATCGAGGATATGATTCTGCTGGAATTTTTGTATCTGA<br>TGGTCGAACTTCTAATTTAGTAAAATCTGTAGGAAGAATTGCTGATTTACGATCTAAAATTGGAATT<br>GATGTTGTTGGTACTACTGGTATTGGACATACTCGATGGGCTACTCATGGACAAGCTACTGAAGA<br>AAATGCTCATCCTCATACTTCTCAAACTGGAAGATTTGTTTTAGTACATAATGGTGTAATTGAAAAT<br>TATTTACAAATGAAAGAACAATATTTAGCTGGACATACTTTTAAAGGACAAACTGATACTGAAATTG<br>CTGTTCATTTAATTGGTGCTTTTGTTGAAGAAGGATGTTCTGTATTAGAAGCTTTTAAAAAAGCTTT<br>ATCTATTATTGAAGGATCTTATGCTTTTGCTTTAATTGATACTGAAGATATGGATACTATTTATGTTGC<br>TAAAAATAAATCTCCTTTATTAATTGGTTTAGGTGAAGGATATAATATGGTATGTTCTGATGCTATGG<br>CTATGATTAGAGAAACTTCTGAATTTATGGAAATTCATGATAAAGAATTAGTTATTTTAACTAAAGAT<br>TCTGCTAGAGTTACTGATTATGCTGGAAATCCTATTGATAGACAATCTTATACTGCTGAATTAGATT<br>TATCTGATATTGGTAAAGGAACTTATCCTTTTTATATGTTAAAAGAAATTGATGAACAACCTACTGTA<br>ATGAGAAGATTAATTTCTACTTATGCTGATGCTAAAGGACAAATGATGATTGATCCTGCTATTATTA<br>GATCTGTACAAGAAGCTGATCGAATTTATATTTTAGCTGCTGGAACTTCTTATCATGCTGGTTTTG<br>CTTCTAAATCTATGTTAGAACATTTAACTGATACTCCTGTAGAATTAGGTGTTGCCTCTGAATGGG<br>GTTATAATATGCCTCTTTTAAGTAAGAAACCAATGTTTATTTTATTATCTCAATCTGGTGAAACTGCT<br>GATTCTCGACAAGTATTAGTTAAGGCAAATGAAATGGGTATTCCTTCTCTTACAGTAACTAATGTTC<br>CTGGATCTACTTTATCTCGAGAAGCTACTTATACCATGCTATTACATGCTGGACCTGAAATTGCTG<br>TAGCTTCTACTAAAGCTTATACTGCTCAAGTTGCTGCTTTAGCTTTCTTAGCAAAGGCTGTAGGTG<br>AAGCTAATGGTAAAAAGGAAGCTATTAGTTTTGATTTAGTTCATGAATTATCTTTAGTTGCTCAATC<br>TATTGAAGCTACACTTTCTGATAAAGAATTAATTGCTGAAAAAGTACAAACTTTATTAGCTACTACT<br>CGAAATGCTTTTTATATTGGACGAGGTAATGATTATTATGTTGCTATGGAAGCTGCTTTAAAATTAA<br>AAGAAATTTCTTATATTCAATGTGAAGGATTTGCTGCTGGAGAATTAAAACATGGTACTATTTCTTT<br>AATTGAAGATGGTACTCCTGTAATTGGTTTAATTTCTTCTTCTGAATTAGTTGCTGCTCATACTCGA<br>GGTAATATTCAAGAAGTAGCTGCTCGAGGTGCTCATGTATTAACTGTAGTAGAAGAAGGTTTAGA<br>AAGAGAAGGAGATGATATTATTATTAATAAAGTTCATCCTTTCTTAGCTCCTATTGCTATGGTAATTC<br>CTACTCAACTTATTGCTTATTATGCTTCTTTACAAAGAGGTTTAGATGTTGATAAACCTCGAAATTTA<br>GCTAAAGCTGTTACTGTTGAATAAtttttcatgatgtttatgtgaatagcataa
glmM	tttttcatgatgtttatgtgaatagcataaacatcgtttttatttttatggtgtttaggttaaatacctaaacatcattttacatttttaaaattaagttctaaa<br>gttatcttttgtttaaatttgcctgtctttataaattacgatgtgccagaaaaataaaatcttagctttttattatagaatttatctttatgtattatattttata<br>agtaataaaaggactgtgctatcttacagcattagttactaattcatgatctggcatttttttggtacgaggaacttatcATGGGTAAATATT<br>TTGGAACTGATGGTGTTAGAGGAGAAGCTAATGTAGAATTAACTCCTGAATTAGCTTTTAAATTAG<br>GTAGATTTGGTGGTTATGTATTATCTCAACATGAAACTGAACGACCTAGAGTATTTGTAGCTCGAG<br>ATACTAGAATTTCTGGTGAAATGTTAGAAGCTGCTTTAATTGCTGGATTATTATCTGTAGGTATTGA<br>AGTTTATAAATTAGGAGTTTTAGCTACTCCTGGTGTTTCTTATTTAGTACGAACTGAAAAAGCTTCT<br>GCTGGAGTTATGATTTCTGCTTCTCATAATCCTGCTTTAGATAATGGAATTAAATTTTTTGGTTCTG<br>ATGGATTTAAATTAGCTGATGAACAAGAATTAGAAATTGAAGCTTTATTAGATGCTAAAGAAGATTT<br>ATTACCTCGACCTTCTGCTGAAGGATTAGGTGCTTTAGTAGATTATCCTGAAGGATTAAGAAAATA<br>TGAAAGATTTTTAGTTACTACTGGTGCTGATTTAGATGGATTAAAAATTGCTTTAGATACTGCTAAT<br>GGAGCTGCTTCTGTTTCTGCTAGAAATGTTTTTTTAGATTTAAATGCTGATATTACTGTAATTGGTG<br>AAAATCCTAATGGTTTAAATATTAATGATGGAATTGGTTCTACTCATCCTGAAAAATTACAAGAATTA<br>GTAACTGAAACTGCTTCTGATATTGGTTTAGCTTTTGATGGTGATTCTGATCGATTAATTGCTGTAG<br>ATGAAAATGGTGCTATTGTAGATGGAGATAAAATTATGTTTATTATTGGTAAATATTTATCTGAAAAA<br>GGATTATTAGCTAAAAATACTATTGTAACTACTGTAATGTCTAATTTAGGTTTTCATAAAGCTTTAGA<br>TTCTTGTGGAATTCATAAAAAAGTAACTGCTGTAGGTGATAGATATGTTGTAGAAGAAATGCGACA<br>ATTTGGTTATAATTTAGGAGGAGAACAATCTGGTCATGTAATTATTATGGATTATAATACTACTGGTG<br>ATGGTCAATTAACTGCTGTTCAATTAACTAAAATTATGAAAGAAACTGGAAAACCTTTATCTGAATT<br>AGCTTCTGAAGTAACTATTTATCCTCAAAAATTAGTTAATATTCGAGTAGATAATTCTATGAAAGAAA<br>GAGCTATGGAAGTACCTGCTATTGCTGATATTATTGCTCAAATGGAAAAAGAAATGTCTGGAAATG<br>GTAGAATTTTAGTTCGACCTTCTGGAACTGAACCTTTATTAAGAGTAATGGCTGAAGCTCCTTCTA<br>ATGAAGAAGTAGATTATTATGTTGATACTATTGCTGCTGTTGTTCGAGCTGAAATTGGTTTAGATTA<br>Aatcttttaattaagtaggaactcggtatat

glmU	caacagatctcaaagttgtagggagggatggtacATGAAAAATTATGCTATTATTTTAGCTGCTGGAAAAGGAACT<br>CGAATGAATTCTGGTTTACCTAAAGTATTACATAAAGTATCTGGTTTATCTATGTTAGAACATGTATT<br>AAAATCTGTTTCTGCTTTAGCTCCTCAAAAACAATTAACTGTTATTGGACATCAAGCTGAACAAGT<br>ACGAGCTGTTTTAGGAGATCAATTATTAACTGTTGTACAAGAAGAACAATTAGGAACTGGACATGC<br>TGTAATGATGGCTGAAGAAGAATTATCTGGTTTAGAAGGTCAAACTTTAGTTATTGCTGGTGATAC<br>TCCTTTAATTCGAGGTGAATCTTTAAAAGCTTTATTAGATTATCATATTCGAGAAAAAAATGTTGCTA<br>CTATTTTAACTGCTAATGCTAAAGATCCTTTTGGATATGGTCGAATTATTAGAAATGCTGCTGGTGA<br>AGTAGTTAATATTGTTGAACAAAAAGATGCTAATGAAGCTGAACAAGAAGTAAAAGAAATTAATACT<br>GGAACTTATATTTTTGATAATAAAAGATTATTTGAAGCTTTAAAACATTTAACTACTGATAATGCTCA<br>AGGAGAATATTATTTAACTGATGTTATTTCTATTTTTAAAGCTTCTCAAGAAAAAGTAGGTGCTTATT<br>TATTAAAAGATTTTGATGAATCTTTAGGAGTTAATGATAGATTAGCTTTAGCTCAAGCTGAAGTAATT<br>ATGCAAGAAAGAATTAATAAACAACATATGTTAAATGGAGTTACTTTACAAAATCCTGCTGCTACTT<br>ATATTGAATCTTCTGTTGAAATTGCTCCTGATGTATTAATTGAAGCTAATGTAACTTTAAAAGGACA<br>AACTCGAATTGGATCTAGATCTGTTATTACTAATGGATCTTATATTTTAGATTCTAGATTAGGAGAAG<br>GAGTAGTAGTTTCTCAATCTGTAATTGAAGGTTCTGTTTTAGCTGATGGAGTAACTGTAGGACCTT<br>ATGCTCATATTCGACCTGATTCTCAATTAGATGAATGTGTTCATATTGGAAATTTTGTAGAAGTAAA<br>AGGATCTCATTTAGGAGCTAATACTAAAGCTGGACATTTAACTTATTTAGGTAATGCTGAAATTGGT<br>TCTGAAGTAAATATTGGAGCTGGTTCTATTACTGTTAATTATGATGGACAACGAAAATATCAAACTG<br>TTATTGGTGATCATGCTTTTATTGGTTCTCATTCTACTTTAATTGCTCCTGTAGAAGTTGGTGAAAA<br>TGCTTTAACTGCTGCTGGTTCTACTATTGCTCAATCTGTTCCTGCTGATTCTGTTGCTATTGGTCG<br>ATCTAGACAAGTAGTAAAAGAAGGTTATGCTAAACGATTACCTCATCATCCTGATCAACCTCAATA<br>Atttttttttaaactaaaataaatctggttaaccatacctggt
hasB	tctggttaaccatacctggtttattttagtttatacacacttttcatatatatatacttaatagctaccataggcagttggcaggacgtccccttacggg<br>acaaatgtatttattgttgcctgccaactgcctaatataaatattagtggacgtcccctgactgtgctatcttacagcattggtagttcgatcgtgga<br>atttctttttttggtacgaggaacttatcATGAAAATTTCTGTAGCAGGCTCAGGATATGTCGGCCTATCCTTGAG<br>TATTTTACTGGCACAACATAATGACGTCACTGTTGTTGATATTATTGATGAAAAGGTGAGATTGATC<br>AATCAAGGCATATCTCCAATCAAGGATGCTGATATTGAGGAGTATTTAAAAAATGCGCCGCTAAAT<br>CTCACAGCGACCCTTGATGGCGCAAGCGCTTATAGCAATGCAGACCTTATTATCATTGCTACTCC<br>GACAAATTATGACAGCGAACGCAACTACTTTGACACAAGGCATGTTGAAGAGGTCATTGAGCAG<br>GTCCTAGACCTAAATGCGTCAGCAACCATTATTATCAAATCAACCATACCACTAGGCTTTATCAAG<br>CATGTTAGGGAAAAATACCAGACAGATCGTATTATTTTTAGCCCAGAATTTTTAAGAGAATCAAAA<br>GCCTTATACGATAACCTTTACCCAAGTCGGATCATTGTTTCTTATGAAAAGGACGACTCACCAAG<br>GGTTATTCAGGCTGCTAAAGCCTTTGCTGGTCTTTTAAAGGAAGGAGCCAAAAGCAAGGATACT<br>CCGGTCTTATTTATGGGCTCACAGGAGGCTGAGGCGGTCAAGCTATTTGCGAATACCTTTTTGG<br>CTATGCGGGTGTCTTACTTTAATGAATTAGACACCTATTCCGAAAGCAAGGGTCTAGATGCTCAG<br>CGCGTGATTGAAGGAGTCTGTCATGATCAGCGCATTGGTAACCATTACAATAACCCTTCCTTTGG<br>ATATGGCGGCTATTGCCTGCCAAAGGACAGCAAACAGCTGTTGGCAAATTATAGAGGCATTCCC<br>CAGTCCTTGATGTCAGCGATTGTTGAGTCCAACAAGATACGAAAATCCTATTTAGCTGAACAAATA<br>TTAGACAGAGCCTCTAGTCAAAAGCAGGCTGGTGTACCATTAACGATTGGCTTTTACCGCTTGAT<br>TATGAAAAGCAACTCTGATAATTTCCGAGAAAGCGCCATTAAAGATATTATTGATATAATCAACGAC<br>TATGGGGTTAATATTGTCATTTACGAACCCATGCTTGGCGAGGATATTGGCTACAGGGTTGTCAA<br>GGACTTAGAGCAGTTCAAAAACGAGTCTACAATCATTGTGTCAAATCGCTTTGAGGACGACCTAG<br>GAGATGTCATTGATAAGGTTTATACGAGAGATGTCTTTGGAAGAGACTAAgtacctctttttttctagctaaaag<br>aagtgaaat
hasA	tctcaaggcggccgcgaaggagatatagagATGCGAACTTTAAAAAATTTAATTACTGTTGTTGCTTTTTCTATT<br>TTTTGGGTTTTATTAATTTATGTTAATGTATATTTATTTGGTGCTAAAGGTTCTTTATCTATTTATGGTT<br>TTTTATTAATTGCTTATTTATTAGTTAAAATGTCTTTATCTTTTTTTTTATAAACCTTTTAAAGGTCGAG<br>CTGGTCAATATAAAGTTGCTGCTATTATTCCTTCTTATAATGAAGATGCTGAATCTTTATTAGAAACT<br>TTAAAATCTGTTCAACAACAAACTTATCCTTTAGCTGAAATTTATGTTGTAGATGATGGTTCTGCTG<br>ATGAAACTGGTATTAAACGAATTGAAGATTATGTTCGAGATACTGGAGATTTATCTTCTAATGTTATT<br>GTACATAGATCTGAAAAAAATCAAGGAAAACGACATGCTCAAGCTTGGGCTTTTGAACGATCTGA<br>TGCTGATGTTTTTTTAACTGTAGATTCTGATACTTATATTTATCCTGATGCTTTAGAAGAATTATTAAA<br>AACTTTTAATGATCCTACTGTTTTTGCTGCTACTGGTCATTTAAATGTTCGAAATAGACAAACTAAT<br>TTATTAACTCGATTAACTGATATTCGATATGATAATGCTTTTGGAGTAGAACGAGCTGCTCAATCTG<br>TTACTGGAAATATTTTAGTATGTTCTGGTCCTTTATCTGTATATCGACGAGAAGTAGTTGTTCCTAA<br>TATTGATAGATATATTAATCAAACTTTTTTAGGAATTCCTGTTTCTATTGGAGATGATAGATGTTTAAC<br>TAATTATGCTACTGATTTAGGAAAAACTGTTTATCAATCTACTGCTAAATGTATTACTGATGTTCCTG<br>ATAAAATGTCTACTTATTTAAAACAACAAAATAGATGGAATAAATCTTTTTTTAGAGAATCTATTATTT<br>CTGTTAAAAAAATTATGAATAATCCTTTTGTAGCTTTATGGACTATTTTAGAAGTATCTATGTTTATGA

	TGTTAGTTTATTCTGTTGTTGATTTTTTTGTTGGAAATGTTAGAGAATTTGATTGGTTAAGAGTTTTA<br>GCTTTTTTAGTTATTATTTTTATTGTAGCTTTATGTCGAAATATTCATTATATGTTAAAACATCCTTTAT<br>CTTTTTTATTATCTCCTTTTTATGGTGTATTACATTTATTTGTATTACAACCTTTAAAATTATATTCTTTA<br>TTTACTATTCGAAATGCTGATTGGGGTACTCGAAAAAAATTATTATAA<ins>ctcttttaaatttacatgttgtaaaggatt</ins>

Uppercase letters indicate codon-optimized gene sequences for plastid expression. Overlapping sequences for HiFi DNA assembly are shown in underlined lowercase letters. The CrTrbcL-ndhB sRNA sequences are indicated in blue. The CrTpsbA-petB sRNA sequences are indicated in red.

Table S2. List of primers used for cloning, preparation of hybridization probes, and RT-PCR analyses.

Name	Sequence (5'-3')	Purpose
oTricisF	gaacaaaagctggagtaatacgactcactataggg	Cloning
oTricisR	gggcgaattgggtacaaaaattctcttagtgggtatg	Cloning
oDK976	catggatactaataggaattc	Cloning
oDK977	acttaattaaaagatatctatatc	Cloning
oAL53	atataccgagttcctacttaattaaaagat	Cloning
oAL54	tttttcatgatgtttatgtgaatagca	Cloning
oAL57	tttgtttaactttaagaaggagatatagatatg	Cloning
oAL68	agcgcagcgagtcagtgagcgaggaagctttagtagaagctacagca	Cloning
oAL69	ctttaagaaggagatatagatatcctcatacttctcaaactgga	Cloning
oAL70	gctattcacataaacatcatgaaaaattattc	Cloning
oAL88	catccatagttgcctgactcc	Cloning
oAL89	cagtttgagaagtatgaggattcaaccctatctcggtctattct	Cloning
oAL253	ctgctggagaattaaaacatggtac	*glmS* probe
oAL254	taatacgactcactatagggcgaggtttatcaacatctaaacctc	*glmS* probe
oAL255	tgatggtcaattaactgctgttc	glmM probe
oAL256	taatacgactcactatagggccaatttcagctcgaacaacag	*glmM* probe
oAL257	gtaatgctgaaattggttctgaagt	*glmU* probe
oAL258	taatacgactcactataggggaggttgatcaggatgatgagg	*glmU* probe
oAL259	cgattgttgagtccaacaagatac	*hasB* probe
oAL260	taatacgactcactatagggcaatgacatctcctaggtcgt	*hasB* probe
oAL261	gaataatccttttgtagctttatggact	RT-PCR / *hasA* gene
oAL293	taccccaatcagcatttcgaatag	RT-PCR / *hasA* gene
oTAM53†	cctgaggtccttttccaacca	RT-PCR / *ACT4* gene
oTAM54†	ggattccggcagcttccatt	RT-PCR / *ACT4* gene
oBock4	cgccgaagtatcgactca	*aadA* probe
oBock5	tcgcgcttagctggataac	*aadA* probe
oBock104	cccagaaagaggctggccc	*psaB* probe
oBock105	cccaaggggcgggaactgc	*psaB* probe

† Primer sequences obtained from Schmidt and Delaney (2010).

Supporting experimental procedures

Methods S1: Construction of the glmS-glmM-glmU-hasB-hasA operon

The *glmS*, *glmM*, *glmU*, *hasB*, and *hasA* coding sequences from *Streptococcus equi* subs. *zooepidemicus* ATCC 35246 (NC_017582.1) were codon-optimized for plastid gene expression (**Table S1**) and acquired by chemical gene synthesis (Invitrogen GeneArt; GeneCust)**.** The synthetic operon was assembled under the control of the T7 RNA polymerase promoter or the plastid *psbA* promoter by recombination between plasmids pAL4 and pAL5, or between plasmids pAL4 and pAL6. Each pair of plasmids harbored 950 bp of overlapping sequence for homologous recombination. Intermediate constructs derived from pRB98 (Stegemann et al., 2003) were designed to generate the plasmids pAL4, pAL5 and pAL6 (**Figure S1**). The plasmid pDK349 harboring the T7 RNA polymerase promoter was generated from plasmid pDK308 (Agrawal et al., 2020; Strand et al., 2023). The sequences for Pt7, Lg10, *atpA* terminator from *C. reinhardtii*, IEE, *atpI* terminator from *C. reinhardtii*, IEE and *atpH* terminator from *C. reinhardtii* were obtained by gene synthesis (Invitrogen GeneArt; GeneCust), amplified by PCR with primer combinations oTricisF and oTricisR (**Table S2**) and inserted into pDK308 by In-Fusion® HD Cloning (Takara; **Figure S1**). The intermediate plasmid pDK410 harboring the plastid CrpsbA promoter was generated from pDK349. The CrpsbA promoter from plasmid pDK325 (Kössler et al., 2021) was amplified by PCR with primer combinations oDK976 and oDK977 (**Table S2**) and cloned into pDK349 following digestion with EcoRI and EcoRV (**Figure S1**).

For construction of pAL4, plasmid pDK410 was digested with the restriction enzyme Acc65I, followed by ligation to the *glmU* and *hasB* synthetic sequences. The resulting plasmid was linearized by EcoRV digestion, and then ligated to the *glmM* sequence and the *glmS* fragment (**Figure S2**) that had been amplified by PCR with the primer combinations oAL53-oAL54 and oAL69-oAL70 (**Table S2**), respectively. The resulting construct was digested with EcoICRI and ligated to the *hasA* gene. Finally, the left border of pDK410 (comprising the homology region from the plastid genome and the *psbA* promoter) was removed by digestion with EcoRV and AhdI, and replaced by the *amp* gene amplified with primers oAL88-oAL89 (**Table S2**). The resulting plasmid pAL4 contains only the right flanking sequence for insertion into the plastid genome by homologous recombination (**Figure S2**). For construction of pAL5, the 5' segment of *glmS* was PCR amplified using primers oAL57 and oAL68 (**Table S2**), and cloned into plasmid vector pDK349 [harboring the T7 RNA polymerase promoter (Pt7) and the 5' UTR of *gene10* from coliphage T7] following digestion

with the restriction enzymes EcoRV and SapI. For construction of pAL6, the 5’ segment of *glmS* was amplified using primers oAL57 and oAL68, and introduced into plasmid pDK410 (harboring the chloroplast *psbA* promoter from *Chlamydomonas reinhardtii* and the 5’ UTR of *gene10* from phage T7) after digestion with EcoRV and SapI. Both pAL5 and pAL6 contain only the left flanking plastid sequence for homologous recombination (**Figure S2**).

Methods S2: Protein extraction and quantification by LC-MS/MS

Protein extraction was conducted according to Sandoval-Ibáñez et al. (2022). Leaf samples were ground in liquid nitrogen and extracted with TKMES buffer [100 mM Tricine-KOH pH 7.5, 10 mM KCl, 1 mM $MgCl_2$, 1 mM EDTA, 10% (w/v) sucrose, 0.2% (v/v) Triton X-100, 1 mM DTT, 2 x protease inhibitor cocktail (cOmplete Protease Inhibitor Cocktail EDTA-free, Roche)].

Total protein extracts were quantified using the Pierce™ Coomassie (Bradford) Protein Assay Kit, and samples of 40 µg were resolved in the first 1.5 cm of an SDS-polyacrylamide gel. The gel pieces were subjected to in-gel trypsin digestion according to Shevchenko et al. (2006) with modifications. Gel pieces were de-stained by three washes with de-staining solution [50% (v/v) acetonitrile (ACN), 50 mM NH_4HCO_3] until the blue dye was no longer visible. The gel pieces were dehydrated with 100% (v/v) ACN, rehydrated with reducing solution (10 mM DTT, 50 mM NH_4HCO_3), and incubated at 56 °C for 30 min. Subsequently, the gel pieces were dehydrated with 100% (v/v) ACN, rehydrated with alkylation solution (55 mM iodoacetamide, 50 mM NH_4HCO_3), and incubated in the dark for 30 min. Finally, the gel pieces were dehydrated with 100% (v/v) ACN, rehydrated with protein digestion solution [10 µg mL^{-1} trypsin/LysC (Promega), 0.001% (v/v) trifluoroacetic acid (TFA), 50 mm NH_4HCO_3], and incubated overnight at 37 °C. For each sample, tryptic peptides were extracted from the gel pieces using 30% (v/v) ACN, 1% (v/v) TFA, and subsequently with a 100% (v/v) ACN, and collected in a single tube. The tryptic peptides were dried in a vacuum centrifuge at 37 °C, dissolved in 0.1% (v/v) TFA, desalted by C18 ZipTip columns (Merck Millipore), and dried again in a vacuum centrifuge at 37 °C. The peptides were finally resuspended in 50 µL of 4% (v/v) ACN, 0.1% (v/v) formic acid (FA).

Proteomic analyses were conducted according to Rolo et al. (2024). Briefly, 3 µL of tryptic peptide samples were processed by liquid chromatography (ACQUITY M-Class (Waters)) coupled to tandem mass spectrometry (Q Exactive Plus; Thermo Scientific) (LC-MS/MS). The two LC solvents A and B were 0.1% (v/v) FA in water and 0.1% (v/v) FA) in ACN,

respectively. Peptides were trapped for 1.5 min with a mobile phase of 4% solvent B at a flow rate of 10 µL min^{-1} and were then resolved at a flow rate of 300 nL min^{-1} with a linear gradient of 4% to 35% solvent B for 30 min, followed by a 2 min gradient from 35% to 80% solvent B, and a 1 min gradient from 80% to 96% solvent B. The column was washed by constant 96% solvent B for 3 min, before re-equilibration at 4% solvent B for 15 min. Precursor ions were selected in the 200 to 2,000 mass to charge (m/z) range at a resolution of 70,000 (at m/z = 200). Ions with single or ≥5 charge states were excluded. The automatic gain control (AGC) target was set to 100,000. Product ions were analyzed at a resolution of 17,500 (at m/z = 200) by data-dependent acquisition.

Protein identification and quantification were performed with MaxQuant version 1.6.0.13 (Cox and Mann 2008). An in-house database including the *Nicotiana tabacum* protein list containing the protein sequences of GlmS, GlmM, GlmU, HasB, and HasA was employed to identify the peptides. The settings to identify the peptide were as follow: protease set to Trypsin/P with a maximum of two missed cleavages, carbamidomethylation of cysteine set as fixed modification, methionine oxidation and protein N-terminal acetylation set as variable modifications, up to five modifications per peptide, and activated label-free quantification (LFQ). All other parameters were the default settings of the software.

The Perseus software version 2.1.3.0 (Tyanova et al. 2016) was used for statistical analysis and processing of LFQ intensity data. LFQ values were transformed with a log_2 function and the peptides belonging to potential contaminants, proteins only identified by single peptides and reverse sequences were removed. The three technical replicates were assigned into an individual sample group, and proteins without a valid LFQ value in at least two of the three technical replicates in at least one sample group were removed from the matrix. Missing intensity values were imputed from the normal distribution of the total matrix using the default parameters. Relevant changes in protein accumulation were selected by significance (*P*-value < 0.05, two-sample Student's t-test) and a greater or equal to 2-fold change of LFQ abundance, comparing the transplastomic lines to the WT under the same cultivation conditions.

Methods S3: Hexose phosphate and UDP-glucose extraction and targeted quantification by LC-MS/MS.

Fructose-6-phosphate (F6P), glucose-6-phosphate (G6P), UDP-glucose (UDP-Glc) and glucose-1-phosphate (G1P) were quantified according to Arrivault et al. (2009, 2015).

Frozen ground aliquots of 15 mg fresh weight (FW) were vigorously mixed with an ice-cold solution of 250 µL chloroform/methanol (3:7; v/v). After incubation at -20 °C for 2 h, the water-soluble metabolites were extracted by adding 400 µL ultrapure water and centrifugation at 21,000 *g* for 10 min at 4 °C. The upper aqueous phase was transferred to a new tube, and the samples were subjected to two additional extractions with 400 µL ultrapure water and centrifugation at 21,000 *g* for 10 min at 4 °C. The second and third aqueous methanolic phases were combined with the first one, frozen in liquid nitrogen, and lyophilized overnight. The dry samples were resuspended in 250 µL ultrapure water, and high molecular mass components were removed by filtration in a 10 kDa Multiscreen Ultracel-10 (Millipore) filter at 2,300 *g* for 2–3 h at 14 °C.

Targeted quantification was performed with a Dionex HPLC system coupled to a Finnigan TSQ Quantum Discovery MS-Q3 (Thermo Scientific) equipped with an electrospray (ESI) interface, operated in the negative ion mode with selected reaction monitoring (SRM), an ion spray voltage of 4000 V and a capillary temperature of 320 °C. Sheath and auxiliary gases were set to 30 and 5 U (arbitrary units), respectively. The argon collision gas pressure was set to 1.1 mTorr, and the quadrupole 1 and quadrupole 3 peak widths were 0.7 m/z. Chromatographic separation was performed with standards of known amounts (to obtain calibration curves) and extracts diluted 1:10. Authentic standards and extracts (100 µl) were passed through a Gemini (C18) 4 x 2.00 mm pre-column (Phenomenex), before separation on a Gemini (C18) 150 x 2.00 mm inner diameter, 5 µm 110 A˚ particle column (Phenomenex) at 35 °C, using a multi-step gradient with online-degassed eluent A [10 mM tributylamine aqueous solution, adjusted to pH 4.95 with 15 mM acetic acid; 5% (v/v) methanol] and eluent B [100% (v/v) methanol]: 0–5 min, 100% A; 5–15 min, 95–90% A; 15–22 min, 90–85% A; 22–37 min, 85–80% A; 37–40 min, 80–65% A, and maintained for 3 min; 43–47 min, 65–40% A, and maintained for 3 min; 50 min, 10% A, and maintained for 4 min; 54 min, 100% A, and maintained for 11 min. The flow rate was 0.2 mL min^{-1} for 0–15 min and 54–65 min, and 0.3 ml min^{-1} for 15–54 min. Prior to injection, a mixture of stable isotopically labelled compounds of known concentrations was added to the standards and extracts to correct for matrix effects (Arrivault et al. 2015). LC-MS/MS SRM peaks were integrated using the ThermoFinnigan processing software package LCQuan-2.5. Metabolites were quantified by comparing the integrated signal peak area with the calibration curves obtained with authentic standards.

Methods S4: Metabolite profiling analyses by GC-MS (GC-EI/TOF-MS)

Equal volumes of residual samples after metabolite-targeted quantifications by LC-MS/MS were dried and analyzed by a gas chromatography-mass spectrometry (GC-MS) method designed for the multi-targeted profiling of polar metabolite fractions that are enriched in primary and small specialized metabolites as described by Fiehn et al. (2000) with modifications reported by Erban et al. (2020). Chemical derivatization for GC analyses and MS measurements were performed as detailed previously (Erban et al. 2020). Methoxyamination was performed by adding 40 µL of 40 mg mL^{-1} methoxyamine hydrochloride and 5 mg mL^{-1} 4-(dimethylamino)pyridine dissolved in pyridine to the dry samples. Samples were vigorously agitated and kept at 30°C for 90 min. Subsequently, 70 µL of N,O-bis(trimethylsilyl)trifluoroacetamide were added for trimethylsilyation and 10 µL of pyridine with an alkane mixture, each at 0.44 mg mL^{-1}. Alkanes served as retention index (RI) markers (Erban et al. 2020). Silylation was 30 min at 37°C. GC-MS was performed by split (split flow ratio, 1:30) and split-less injection at 270°C into a GC-electron impact ionization/ time-of-flight-MS (GC-EI/TOF-MS) system of an Agilent 6890N24 gas chromatograph (Agilent Technologies) hyphenated to a Pegasus III time-of-flight mass spectrometer (LECO Instrumente GmbH). A 5% (v/v) phenyl–95% (v/v) dimethylpolysiloxane DB-5 fused silica capillary column of 30 m length, 0.25 mm inner diameter, 0.25 µm film thickness with an integrated 10 m precolumn (Agilent Technologies) was operated at constant flow of 0.6 mL min^{-1}. The temperature program comprised an initial 1 min isothermal phase at 70°C, a ramp to 350°C at a rate of 9°C min^{-1} and a 5 min phase at 350°C. The mass spectrometer was set to EI at 70 eV, and a scan range of 70 – 600 amu with a scan rate of 20 spectra s^{-1}. Acquired GC-EI/TOF-MS data were smoothed, baseline corrected and exported to CDF-files using ChromaTOF software (Version 4.22; LECO). RI alignment and peak-height picking were done with the TagFinder software (Luedemann et al. 2008). Metabolite annotation was performed manually supervised by matching mass spectra and RIs to the reference data of the Golm Metabolome Database (Hummel et al. 2013). Abundance data of arbitrary units were normalized by sample fresh weight (mg FW) and maximum scaled (%) per analytical feature prior to statistical procedures. Relevant changes in metabolite accumulation were selected by significance (*P*-value < 0.05, two-sample Student's t-test) comparing the transplastomic lines to the wild type under the same cultivation conditions. Heatmaps were generated by the Perseus software version 2.1.3.0 (Tyanova et al. 2016).

Supporting References

Agrawal S, Karcher D, Ruf S, Bock R (2020) The Functions of Chloroplast Glutamyl-tRNA in Translation and Tetrapyrrole Biosynthesis. *Plant Physiology* **183**: 263–276.

Arrivault S, Guenther M, Fry SC, Fuenfgeld MMFF, Veyel D, Mettler-Altmann T, Stitt M, Lunn JE (2015) Synthesis and Use of Stable-Isotope-Labeled Internal Standards for Quantification of Phosphorylated Metabolites by LC-MS/MS. *Analytical Chemistry* **87**: 6896–6904.

Arrivault S, Guenther M, Ivakov A, Feil R, Vosloh D, van Dongen JT, Sulpice R, Stitt M (2009) Use of reverse-phase liquid chromatography, linked to tandem mass spectrometry, to profile the Calvin cycle and other metabolic intermediates in Arabidopsis rosettes at different carbon dioxide concentrations. *Plant Journal* **59**; 826–839.

Cox J, Mann M (2008) MaxQuant enables high peptide identification rates, individualized p.p.b.-range mass accuracies and proteome-wide protein quantification. *Nature Biotechnology* **26**: 1367–1372.

Erban A, Martinez-Seidel F, Rajarathinam Y, Dethloff F, Orf I, Fehrle I, Alpers J, Beine-Golovchuk O, Kopka J (2020) Multiplexed Profiling and Data Processing Methods to Identify Temperature-Regulated Primary Metabolites Using Gas Chromatography Coupled to Mass Spectrometry. *Methods in Molecular Biology* **2156**: 203–239.

Fiehn O, Kopka J, Dörmann P, Altmann T, Trethewey RN, Willmitzer L (2000) Metabolite profiling for plant functional genomics. *Nature Biotechnology* **18**: 1157–1161.

Hoelscher MP, Forner J, Calderone S, Krämer C, Taylor Z, Loiacono FV, Agrawal S, Karcher D, Moratti F, Kroop X, Bock R (2022) Expression strategies for the efficient synthesis of antimicrobial peptides in plastids. *Nature Communications* **13**: 5856.

Hummel J, Strehmel N, Bölling C, Schmidt S, Walther D, Kopka J (2013) Mass Spectral Search and Analysis Using the Golm Metabolome Database. In: *The Handbook of Plant Metabolomics* (Weckwerth W, Kahl G, eds), pp 321–343. John Wiley & Sons, Ltd: Oxford, UK. doi: 10.1002/9783527669882.ch18

Kössler S, Armarego-Marriott T, Tarkowská D, Turečková V, Agrawal S, Mi J, Souza LP de, Schöttler MA, Schadach A, Fröhlich A, Bock R, Al-Babili S, Ruf S, Sampathkumar A, Moreno JC (2021) Lycopene β-cyclase expression influences plant physiology, development, and metabolism in tobacco plants. *Journal of Experimental Botany* **72**: 2544–2569.

Luedemann A, Strassburg K, Erban A, Kopka J (2008) TagFinder for the quantitative analysis of gas chromatography—mass spectrometry (GC-MS)-based metabolite profiling experiments. *Bioinformatics* **24**: 732–737.

Rolo D, Sandoval-Ibáñez O, Thiele W, Schöttler MA, Gerlach I, Zoschke R, Schwartzmann J, Meyer EH, Bock R (2024) CO-EXPRESSED WITH PSI ASSEMBLY1 (CEPA1) is a photosystem I assembly factor in Arabidopsis. *Plant Cell* **36**: 4179–4211.

Sandoval-Ibáñez O, Rolo D, Ghandour R, Hertle AP, Armarego-Marriott T, Sampathkumar A, Zoschke R, Bock R (2022) De-etiolation-induced protein 1 (DEIP1) mediates assembly of the cytochrome b6f complex in Arabidopsis. *Nature Communications* **13**: 4045.

Schmidt GW, Delaney SK (2010) Stable internal reference genes for normalization of real-time RT-PCR in tobacco (*Nicotiana tabacum*) during development and abiotic stress. *Molecular Genetics and Genomics* **283**: 233–241.

Shevchenko A, Tomas H, Havlis J, Olsen JV, Mann M (2006) In-gel digestion for mass spectrometric characterization of proteins and proteomes. *Nature Protocols* **1**: 2856–2860.

Stegemann S, Hartmann S, Ruf S, Bock R (2003) High-frequency gene transfer from the chloroplast genome to the nucleus. *Proceedings of the National Academy of Sciences of the United States of America* **100:** 8828–8833.

Strand DD, Karcher D, Ruf S, Schadach A, Schöttler MA, Sandoval-Ibañez O, Hall D, Kramer DM, Bock R (2023) Characterization of mutants deficient in N-terminal phosphorylation of the chloroplast ATP synthase subunit β. *Plant Physiology* **191**: 1818–1835.

Tyanova S, Temu T, Sinitcyn P, Carlson A, Hein MY, Geiger T, Mann M, Cox J (2016) The Perseus computational platform for comprehensive analysis of (prote)omics data. *Nature Methods* **13**: 731–740.
